# Integrative analysis of adenosine-related RNA modifications defines molecular subtypes of bladder cancer and identifies CES1 as a driver of tumor progression and immunotherapy resistance

**DOI:** 10.1186/s12885-026-15897-4

**Published:** 2026-03-25

**Authors:** Xingxing Huo, Jian Qi, Yongfu Zhu, Hang Song, Wei Han, Shujie Wang

**Affiliations:** 1 Science Island Branch of Graduate School, University of Science and Technology of China, Hefei, 230031 China; 2 Anhui Province Key Laboratory of Medical Physics and Technology, Institute of Health and Medical Technology, Hefei Institutes of Physical Science, Chinese Academy of Sciences, Hefei, 230031 China; 3 Experimental Center of Clinical Research, The First Affiliated Hospital of Anhui University of Chinese Medicine, Hefei, Anhui 230031 China; 4 Department of Oncology, The First Affiliated Hospital of Anhui University of Chinese Medicine, Hefei, 230031 China; 5 School of Integrated Chinese and Western Medicine, Anhui University of Chinese Medicine, Hefei, 230012 China

**Keywords:** Bladder cancer, RNA modification, “Writer” genes, Mutation landscape, Tumor microenvironment, Immunotherapy, Prognostic model, CES1

## Abstract

**Background:**

Adenosine-related RNA modifications, including N6-methyladenosine (m^6^A), N1-methyladenosine (m^1^A), alternative polyadenylation (APA), and adenosine-to-inosine (A-to-I) editing, critically regulate transcriptional programs in cancer. However, their coordinated contribution to bladder cancer biology and response to immunotherapy remains poorly understood.

**Methods:**

We systematically characterized four classes of RNA modification “writer” genes in bladder cancer, assessing somatic mutations, copy-number variations, and mRNA expression patterns across seven independent cohorts, including TCGA-BLCA, E-MTAB-4321, and GEO datasets (GSE32894, GSE13507, GSE48276, GSE70691, and GSE69795). Unsupervised consensus clustering identified RNA modification-based molecular subtypes, which were used to construct an RNA modification-based prognostic and immunotherapy response score (RMP_Score). This score was validated across multiple external cohorts, including the IMvigor210 anti-PD-L1 dataset. Differentially expressed genes (DEGs) between subtypes were assessed and integrated through Kaplan-Meier survival analysis and Cox proportional hazards regression to construct the RMP_Score. Bulk and single-cell transcriptomic analyses, complemented by in vitro functional assays, were performed to investigate the role of carboxylesterase 1 (CES1), a key component of the RMP_Score. Tumor microenvironment (TME) composition was quantified using single-sample gene set enrichment analysis (ssGSEA).

**Results:**

RNA modification “writer” genes exhibited frequent somatic mutations and copy-number amplifications, which were strongly associated with transcriptional dysregulation and inferior overall survival. Unsupervised clustering revealed two distinct subtypes: a metabolism-enriched cluster associated with favorable prognosis, and an immune- and stroma-enriched cluster characterized by abundant regulatory T-cell infiltration and adverse clinical outcomes. The RMP_Score robustly stratified patient survival across six independent cohorts, correlated with differential sensitivity to inhibitors targeting the JNK/p38, PI3K/mTOR, and WNT pathways, and accurately predicted clinical responses to anti-PD-L1 immunotherapy, with low RMP_Scores demonstrating superior responses. CES1 emerged as a robust predictor of poor survival and immunotherapy resistance, closely associated with tumor progression, extracellular matrix remodeling, and an immunosuppressive, fibroblast-rich TME. Functionally, CES1 silencing significantly attenuated bladder cancer cell viability, invasive capacity, and migratory potential in vitro.

**Conclusions:**

These findings established an RNA modification-based framework for molecular classification and risk stratification in bladder cancer, and identified CES1 as a metabolically driven regulator of the TME with potential utility as a prognostic and predictive biomarker for immune checkpoint blockade.

**Supplementary Information:**

The online version contains supplementary material available at 10.1186/s12885-026-15897-4.

## Introduction

Bladder cancer is among the most common and aggressive malignancies of the urinary system worldwide. According to recent Global Cancer Statistics, it accounts for approximately 573,000 new cases and 213,000 deaths annually. The incidence increases sharply after age 65 and is three to four times more frequent in men than in women [1–3]. Despite advances in surgical techniques, chemotherapy, and other multimodal treatment strategies, clinical outcomes for patients with advanced bladder cancer remain poor [4].

Recent studies have further expanded the mechanistic landscape of bladder cancer, particularly in relation to treatment resistance and immune evasion, including ferroptosis-resistance programs [5, 6], altered interferon signaling [7], lipid- and inflammation- associated activation of the Janus kinase (JAK)- signal transducer and activator of transcription (STAT) pathway [8], and WNT/β-catenin-driven proliferative programs [9]. Single-cell and epigenomic analyses have also refined models of tumor evolution and therapy-associated cell-cell communication [10, 11]. Spatial and single cell atlases further reveal convergent features within metastatic microenvironments and clinically relevant fibroblast programs [12]. In addition, immune-metabolic predictors such as indoleamine 2,3-dioxygenase 1 (IDO1) have been proposed for high-risk non-muscle-invasive bladder cancer (NMIBC) [13], and metabolic axes, such as folate-linked AMP-activated protein kinase (AMPK) mechanistic target of rapamycin (mTOR) signaling, have been implicated in malignant progression [14]. Despite these advances, there remains a practical need for integrated biomarkers capable of capturing both tumor-intrinsic programs and microenvironmental states.

Bladder tumorigenesis is a multistep process, with epigenetic dysregulation playing a central role [15]. Understanding epigenetic mechanisms underlying tumor initiation, progression, and therapeutic response is essential for improving diagnosis, risk stratification, and treatment. Epigenetic regulation encompasses heritable yet reversible mechanisms that control gene expression without altering the underlying DNA sequence. Principal epigenetic modalities include DNA methylation [16], histone modifications [17, 18], and RNA modifications [19]. RNA modifications, collectively known as epitranscriptomics, involve chemical modifications on RNA molecules as well as their associated regulatory proteins. These modifications govern alternative splicing, RNA stability, subcellular localization, and translational efficiency, thereby dynamically reshaping cellular gene-expression programs [20]. To date, more than 100 distinct RNA modifications have been identified, including N6-methyladenosine (m^6^A), N1-methyladenosine (m^1^A), 5-methylcytidine (m^5^C), and 7-methylguanosine (m^7^G) [21, 22]. Importantly, these modifications function within interconnected regulatory networks rather than as isolated molecular events [23, 24].

Among these, m^6^A is the most abundant internal modification in eukaryotic mRNA and is prevalent in non-coding RNAs as well [25, 26]. m^6^A marks are dynamically installed by multicomponent methyltransferase complexes (“writer”), which include METTL3, METTL14, WTAP, KIAA1429, and other associated cofactors [27]. Dysregulated m^6^A modification has been implicated in tumor initiation, proliferation, invasion, metastasis, and immune evasion across multiple malignancies, including bladder cancer [28–32]. Similarly, m^1^A modification modulates RNA structure and function, thereby contributing to post-transcriptional gene regulation [23]. Its writer enzyme complexes, including TRMT61A and TRMT6, have been implicated in tumorigenesis and disease progression [33–36].

Alternative polyadenylation (APA) represents another critical post-transcriptional regulatory mechanism. By generating mRNA isoforms with distinct 3′ untranslated regions (3′UTRs), APA modulates mRNA stability, subcellular localization, and translational efficiency [37]. This process is governed by core processing complexes including CPSF and CSTF, and aberrant APA has been demonstrated to drive oncogene activation and promote cancer progression [38]. In addition, adenosine-to-inosine (A-to-I) RNA editing, catalyzed by the ADAR family of enzymes (ADAR, ADARB1, and ADARB2), is widespread in human cells [39, 40]. A-to-I editing can alter protein coding sequences or alter regulatory elements, thereby affecting cancer cell growth and survival. Its dysregulation has been reported in multiple tumor types [39, 40].

Given the diversity and functional interplay of RNA writer-mediated modifications, understanding how distinct RNA modification systems collectively shape cancer phenotypes is essential. We hypothesize that the writers governing m^6^A, m^1^A, APA, and A-to-I editing constitute an integrated regulatory network in bladder cancer and that systematic dissection of this network will yield novel insights into tumor progression and therapeutic vulnerability.

Immune checkpoint blockade (ICB) targeting PD-1/PD-L1 or CTLA-4 has revolutionized cancer therapy. However, durable responses are achieved in only approximately 20% of patients with solid tumors, and both primary and acquired resistance remain common [41]. Identifying molecular determinants of ICB sensitivity is therefore a major clinical priority [42]. Emerging evidence highlights a close connection between RNA modifications and the tumor immune microenvironment (TIME). For example, METTL3-mediated m^6^A modification promotes dendritic cell activation and T cell priming [43], regulates the function of tumor-infiltrating T cells [44], and drives the immunosuppressive activity of regulatory T cells (Tregs) [45]. In colorectal cancer, depletion of METTL3 or METTL14 enhances responses to anti-PD-1 therapy [46], whereas METTL3-dependent macrophage polarization has been linked to ICB resistance [47]. Notably, most studies have focused on individual regulators, predominantly m^6^A writers, without integrating multiple RNA modification systems. A comprehensive, multi-layered analysis of writer-mediated regulatory patterns therefore provides a more complete framework for understanding immune regulation and guiding personalized immunotherapy.

In parallel, multi-omics integration is increasingly being used to refine prognostication and guide therapy selection across cancers, including bladder cancer and renal cell carcinoma [48–52]. These developments provide a methodological framework for constructing robust, biologically grounded scoring systems that link tumor-intrinsic features with microenvironmental phenotypes and treatment outcomes.

To date, few studies have systematically explored how RNA modification patterns influence bladder cancer progression or immunotherapy response. Here, we integrated transcriptomic and clinical data from seven public cohorts (*n* = 1,696) and focused on genes involved in four RNA modification pathways (m^6^A, m^1^A, APA, and A-to-I editing) (Table S1). Distinct RNA modification patterns defined molecular subtypes, and a scoring model based on these patterns robustly predicts patient prognosis and response to ICB across multiple independent cohorts (E-MTAB-4321, GSE13507, GSE32894, GSE69795, GSE70691, and GSE48276) (Table S2). Its predictive value for anti-PD-L1 therapy was further validated in the IMvigor210 cohort [53]. Through this integrative analysis, carboxylesterase 1 (CES1) emerged as a key candidate gene associated with advanced pathological stage, high tumor grade, and poor outcomes. In vitro experiments confirmed that CES1 promotes cancer cell viability, migration, and invasion. Integrated analyses of bulk and single-cell transcriptomic data, combined with functional validation, suggest that CES1 drives tumor progression by reprogramming lipid metabolism in both tumor cells and cancer-associated fibroblasts (CAFs). This metabolic rewiring facilitates extracellular matrix remodeling and contributes to the establishment of an immunosuppressive tumor microenvironment (TME). Collectively, our findings established an RNA modification-based framework for molecular subtyping and risk stratification in bladder cancer and identified CES1 as a potential biomarker and therapeutic target.

## Materials and methods

### Data acquisition and processing

Transcriptomic and matched clinical data from 1,696 patients with bladder cancer across seven independent cohorts were retrieved from publicly available databases, including Bladder Urothelial Carcinoma in The Cancer Genome Atlas (TCGA-BLCA, UCSC Xena, accessed on 13 April 2024), E-MTAB-4321 (ArrayExpress, accessed on 13 April 2024), and five Gene Expression Omnibus (GEO) datasets (GSE32894, GSE13507, GSE48276, GSE70691, and GSE69795; accessed on 13 April 2024). The TCGA-BLCA cohort designated used as the training set, while the remaining datasets were used for independent validation. For GEO datasets generated on the same microarray platform (GSE48276, GSE70691, and GSE69795), batch effects were corrected using the sva R package. All analyses were performed in R (version 4.1.2) with appropriate Bioconductor packages.

Data from the IMvigor210 cohort, comprising patients with urothelial carcinoma treated with the anti-PD-L1 antibody atezolizumab, were used to assess the association between the RNA modification score and response to ICB. Normalized gene expression profiles and clinical annotations were obtained from the IMvigor210CoreBiologies R package.

### Identification of RNA modification patterns based on “writer” genes

A total of 30 RNA modification “writer” genes were analyzed, including 10 m^6^A writers (METTL3, METTL14, WTAP, KIAA1429, METTL16, ZCCHC4, METTL5, RBM15, ZC3H13, and RBM15B), five m^1^A writers (TRMT10A, TRMT61A, TRMT10C, TRMT61B, and TRMT6), 12 APA-related factors (CPSF1–4, CSTF1, CSTF2, CSTF3, PCF11, CFI, CLP1, NUDT21, and PABPN1), and three A-to-I RNA editing enzymes (ADAR, ADARB1, and ADARB2). The expression profiles of these genes in TCGA-BLCA tumor samples were analyzed through unsupervised consensus clustering using the ConsensusClusterPlus package. We performed 1,000 resampling iterations to identify stable RNA modification patterns.

### Functional characterization of RNA modification patterns

Biological differences between RNA modification patterns were assessed using Gene Set Variation Analysis (GSVA) implemented in the GSVA package. Single-sample gene set enrichment analysis (ssGSEA) was performed with KEGG and HALLMARK gene sets retrieved from the msigdbr package. Functional annotation of the 30 RNA modification writer genes was conducted using the clusterProfiler package.

### Estimation of immune cell infiltration in the TME

The relative abundance of 28 immune cell populations within the TME was estimated using ssGSEA. Log_2_-transformed transcripts per million (TPM) expression matrices, together with previously published gene signatures for the 28 immune cell subsets, were used as input data. ssGSEA was implemented with the parameters kcdf = “Gaussian” and method = “ssgsea”.

### Construction of the RNA modification signature

Differentially expressed genes (DEGs) between RNA modification patterns were identified using the limma package on log_2_(TPM + 1)–transformed expression data. Genes with an adjusted *P* value < 0.005 and an absolute log_2_ fold change > 1.5 were considered significant. The prognostic relevance of DEGs was assessed by Kaplan-Meier survival analysis with log-rank testing, and genes with *P* < 0.005 were retained for further analysis. To reduce the risk of overfitting, least absolute shrinkage and selection operator (LASSO) Cox regression was performed using the glmnet package, and genes with nonzero coefficients were selected. These genes were subsequently included in a multivariable Cox proportional hazards model. Genes that remained independently prognostic (*P* < 0.05) were used to construct the final RNA modification-based prognostic and immunotherapy response score (RMP_Score). For each patient, the RMP_Score was calculated as a weighted linear combination of gene-expression values and their corresponding Cox regression coefficients.

### Association between the RMP_Score and drug sensitivity

Predicted half-maximal inhibitory concentration (IC50) values for 138 anticancer drugs in TCGA-BLCA samples were estimated using the pRRophetic package. The associations between the RMP_Score and drug sensitivity were assessed using Spearman correlation analysis. Correlations with |Rs| > 0.1 and *P* < 0.05 were considered statistically significant.

### Single-cell RNA-sequencing (scRNA-seq) data acquisition and preprocessing

Publicly available scRNA-seq data were obtained from the Tumor Immune Single-cell Hub 2 (TISCH2) database (http://tisch.comp-genomics.org/, accessed on 13 March 2024) [54]. Specifically, dataset GSE130001, comprising tumor samples from patients with muscle-invasive bladder cancer, was analyzed. Cells in this dataset were enriched for CD45- negative (CD45^−^) populations prior to library preparation, enabling focused analysis of non-immune components of the TME.

### Immunohistochemistry (IHC) images from the Human Protein Atlas (HPA)

For the analysis of CES1 protein expression in urothelial carcinoma, IHC images were obtained from the HPA, a widely used resource for protein localization and biomarker discovery. The staining intensity for CES1 was assessed and categorized as negative, low, moderate, or high according to standardized visual evaluation criteria.

### Cell lines and basal gene-expression analysis

Human bladder cancer cell lines (T24, 5637, BT-B, and UM-UC-3), as well as the immortalized normal human urothelial cell line SV-HUC-1, were obtained from the Cell Bank of the Chinese Academy of Sciences (Shanghai, China). Cells were maintained in Roswell Park Memorial Institute (RPMI)-1640 medium supplemented with 10% heat-inactivated fetal bovine serum and 1% penicillin–streptomycin at 37 °C in a humidified incubator with 5% CO₂. Basal expression of CES1 in bladder cancer cell lines, normalized to SV-HUC-1, was quantified by quantitative polymerase chain reaction (qPCR).

### siRNA and plasmid transfection

Cells in the logarithmic growth phase were seeded at a density of 3–5 × 10^4^ cells/mL. Transfections were performed using Lipofectamine 3000 reagent according to the manufacturer’s protocol. For knockdown experiments, BT-B cells were transfected with CES1-specific small interfering RNA (siRNA), with non-targeting scrambled siRNA serving as a negative control. For overexpression studies, the CES1 coding sequence was subcloned into the pCDH-CMV-MCS-EF1-CopGFP-T2A-Puro lentiviral vector (designated pCDH-CMV-CES1). T24 cells were transfected with pCDH-CMV-CES1, with the empty vector as a control. Cells were harvested 48 h post-transfection for subsequent analyses.

### Quantitative real-time PCR (qRT-PCR)

Total RNA was extracted using the TRIzol Plus RNA Purification Kit, and first-strand cDNA was synthesized from 1 µg of RNA using PrimeScript RT Master Mix. qRT-PCR was performed with TB Green Premix Ex Taq II on a StepOnePlus Real-Time PCR System (primer sequences are listed in Table S3). Cycling conditions included an initial denaturation at 95 °C for 30 s, followed by 40 cycles of 95 °C for 5 s and 60 °C for 30 s. Relative gene expression was calculated using the 2^−ΔΔCt^ method, with GAPDH as the internal reference.

### Western blot analysis

Total protein was extracted using radioimmunoprecipitation assay (RIPA) lysis buffer, quantified using the bicinchoninic acid (BCA) assay, separated by sodium dodecyl sulfate-polyacrylamide gel electrophoresis (SDS-PAGE), and transferred onto polyvinylidene fluoride (PVDF) membranes. Membranes were blocked and incubated overnight at 4 °C with primary antibodies against CES1 (1:1,000 dilution) and GAPDH (1:1,000 dilution). Following incubation with HRP-conjugated secondary antibodies, immunoreactive signals were detected by enhanced chemiluminescence. Band intensities were quantified using ImageJ software.

### Cell viability assay

Cell viability was assessed using the Cell Counting Kit-8 (CCK-8) assay. Cells were seeded in 96-well plates at a density of 2.5 × 10^3^ cells per well. At designated time points, 10 µL of CCK-8 reagent was added to each well and incubated for 2 h at 37 °C, followed by spectrophotometric measurement of absorbance at 450 nm.

### Cell apoptosis assay

After transfection or treatment, cells were collected, washed, and stained with Annexin V conjugated with allophycocyanin (Annexin V-APC) and propidium iodide (PI) using the BD Annexin V-APC Apoptosis Detection Kit. The samples were then incubated at room temperature in the dark and analyzed by flow cytometry.

### Cell invasion assay

Cell invasion was assessed using 24-well Matrigel-coated Transwell chambers. Cells (1 × 10^5^) suspended in serum-free medium were seeded into the upper chamber, while medium containing 10% fetal bovine serum was added to the lower chamber as a chemoattractant. After 24 h, non-invading cells were removed, and invading cells on the lower surface of the membrane were fixed, stained with crystal violet, and quantified under a light microscope.

### Wound healing assay

Cells were grown to near confluence in six-well plates, and a linear scratch was created using a sterile pipette tip. After removing debris by washing, serum-free medium was added. Wound closure was imaged at 0, 24, and 48 h, and migration distances were quantified using ImageJ.

### Statistical analysis

All statistical analyses were performed using R (version 4.1.2). Continuous variables were compared using the Wilcoxon rank-sum test for two-group comparisons or the Kruskal-Wallis test for multiple-group comparisons. Survival differences were evaluated using Kaplan-Meier analysis with log-rank testing. All statistical tests were two-tailed, and a *P* value < 0.05 was considered statistically significant.

## Results

### Mutation landscape and expression patterns of adenosine-related RNA modification “writer” genes in bladder cancer

**Fig. 1:**
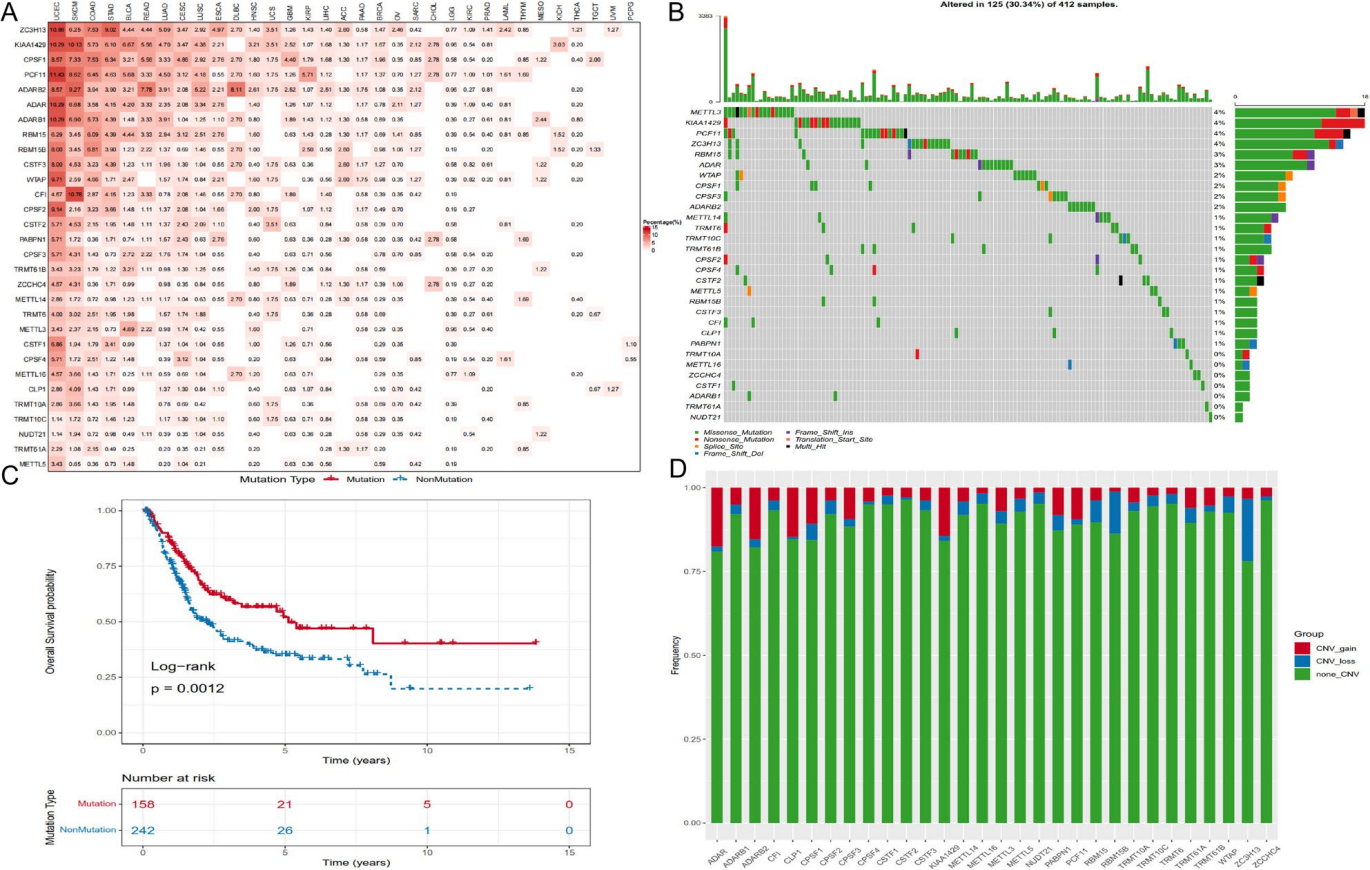
Genomic and transcriptional alterations of RNA modification writer genes in bladder cancer. **A** Mutation frequencies of RNA modification writer genes across 33 TCGA cancer types. Cancer types are shown on the x-axis with sample sizes indicated, and writer genes are listed on the y-axis. **B** Somatic mutation landscape of 30 RNA modification writer genes in the TCGA-BLCA cohort. Each column represents one patient. Numbers on the right indicate mutation frequencies, and the bar plot summarizes mutation types. **C** Kaplan-Meier overall survival curves comparing patients with (red) or without (blue) mutations in writer genes in the TCGA-BLCA cohort. *P* values were calculated using a two-sided log-rank test, with *P* < 0.05 considered statistically significant. **D** Frequencies of copy number gain (CNV_gain, red), copy number loss (CNV_loss, blue), and non-CNV events (green) for each writer gene in the TCGA-BLCA cohort; bar height represents event frequency

To delineate the genetic and transcriptional landscape of adenosine-related RNA modification writer genes in bladder cancer, we systematically analyzed somatic mutations, copy number variations (CNVs), and mRNA expression profiles of 30 writer genes (10 m^6^A, 5 m^1^A, 12 APA, and 3 A-to-I editing) in the TCGA-BLCA cohort. Compared with the TCGA TGCT, PCPG, and UVM cohorts, individual writer gene mutation frequencies were relatively low, whereas the BLCA cohort showed a markedly higher overall mutation burden across RNA modification writer genes (Fig. 1A). Somatic mutations affecting at least one writer gene were detected in 125 bladder cancer samples, accounting for 30.34% of cases (Fig. 1B). Among these genes, METTL3 exhibited the highest mutation frequency (4%), followed by KIAA1429, PCF11, and ZC3H13, whereas mutations in TRMT10A, METTL16, ZCCHC4, CSTF1, ADARB1, TRMT61A, and NUDT21 were rare. Survival analysis revealed that patients harboring mutations in any RNA modification writer gene had significantly shorter overall survival (OS) than those without these alterations (log-rank *P* = 0.0012; Fig. 1C), indicating that genetic perturbations of these epitranscriptomic regulators are clinically relevant and may contribute to bladder cancer progression.

**Fig. 2:**
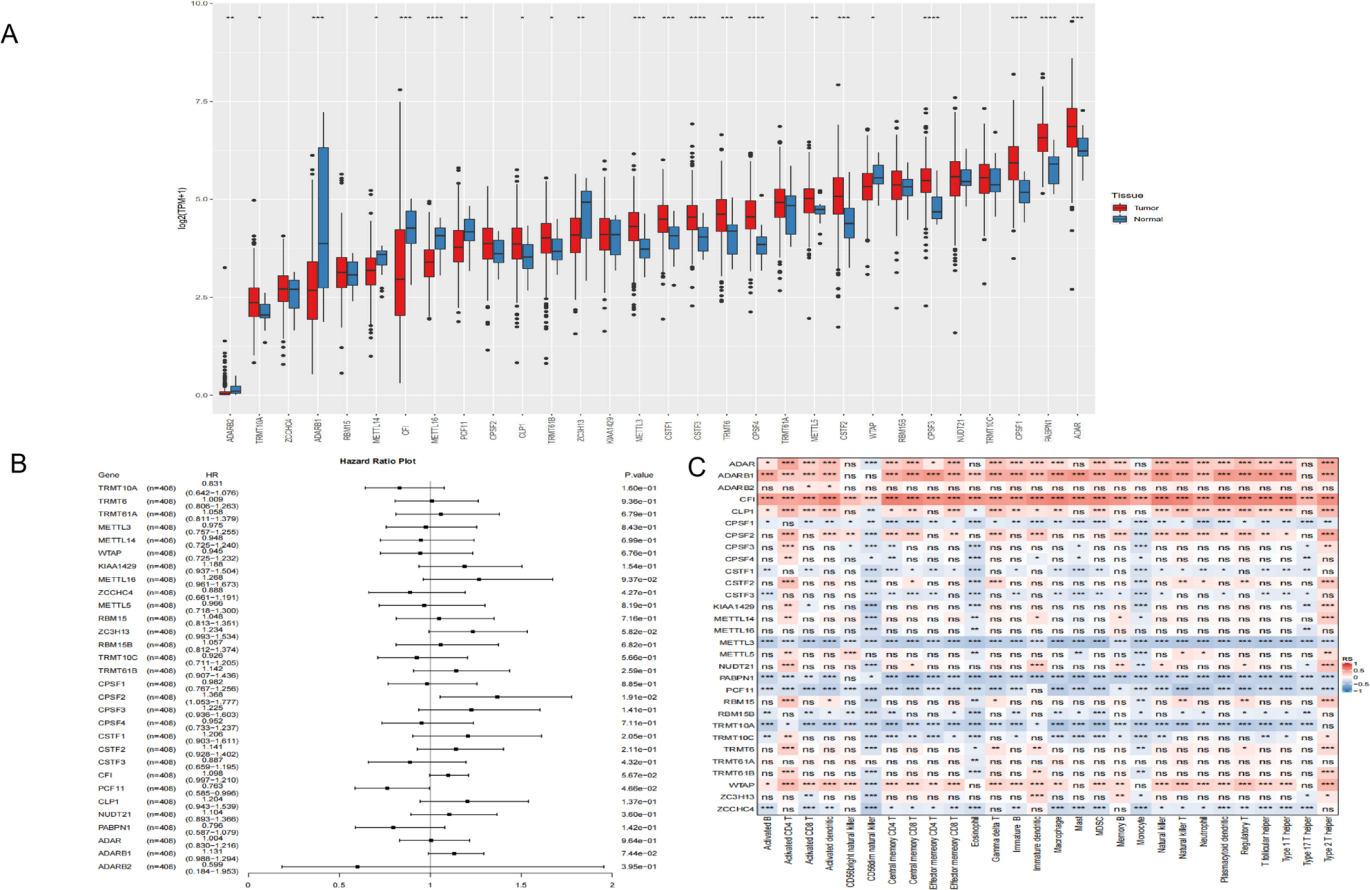
Biological characteristics of RNA modification writer genes. **A** Expression levels of 30 writer genes across four RNA modification types in normal (blue) and bladder cancer (red) tissues. **B** Associations between writer-gene expression and overall survival estimated by univariate Cox regression. **C** Heatmap showing Spearman correlations between tumor microenvironment immune infiltration and the writer-gene expression in bladder cancer. Positive correlations are shown in red and negative correlations in blue. *P* < 0.05, ^**^P < 0.01, ^***^P < 0.001

Analysis of CNV profiles revealed frequent copy number gains in several writer genes, including ADAR, ADARB2, CLP1, and KIAA1429 (Fig. 1D). Consistent with these genomic alterations, comparison of transcript levels between tumor and normal tissues showed that the majority of writer genes were significantly upregulated in bladder cancer (Fig. 2A). Genes with CNV amplification, such as ADAR and CLP1, also had pronounced increases in mRNA expression (Figs. 1D and 2A), supporting copy number gain as an important mechanism underlying writer overexpression.

Univariate Cox regression analysis identified CPSF2 and PCF11 as significantly associated with OS in the TCGA-BLCA cohort (Fig. 2B). Given growing evidence linking RNA modifications to immune regulation within the TME [43, 44], we next examined the relationship between writer-gene expression and immune infiltration. Using ssGSEA, we observed strong correlations between the expression profiles of RNA modification writers and the abundance of multiple immune cell populations within the TME (Fig. 2C), suggesting that these regulators may play a role in shaping tumor–immune interactions.

### Consensus clustering identified distinct RNA modification patterns associated with the TIME

**Fig. 3:**
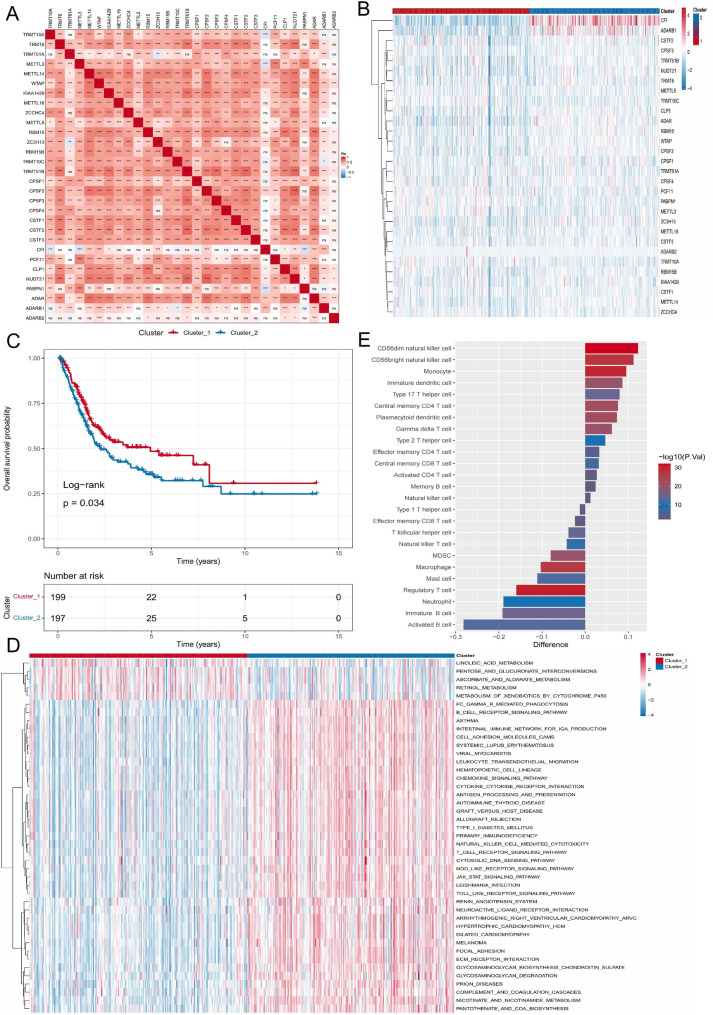
Biological characteristics of distinct RNA modification patterns. **A** Correlation heatmap of RNA modification writer genes in bladder cancer, showing positive (red) and negative (blue) Spearman correlations (^*^P < 0.05, ^**^P < 0.01, ^***^P < 0.001). **B** Unsupervised clustering of bladder cancer samples based on writer-gene expression. **C** Kaplan–Meier overall survival curves for the two RNA modification clusters (Cluster_1, red; Cluster_2, blue). **D** GSVA heatmap showing pathway-activity differences between RNA modification patterns. **E** Relative abundance of tumor-infiltrating immune cells in Cluster_1 and Cluster_2 estimated by ssGSEA

We first examined transcriptional co-regulation among RNA modification writer genes. Correlation analysis revealed that most writers were positively associated with one another, with only a few negative correlations observed (Fig. 3A), suggesting coordinated regulation across different RNA modification pathways.

Unsupervised consensus clustering based on the expression of the 30 writer genes stratified the TCGA-BLCA cohort into two stable RNA modification patterns, designated Cluster_1 and Cluster_2 (Fig. 3B). Survival analysis indicated that patients in Cluster_1 had significantly better OS than those in Cluster_2 (log-rank *P* = 0.034; Fig. 3C). Functional characterization using GSVA revealed pronounced pathway-level differences between the two patterns. Cluster_1 was enriched for metabolic pathways, including linoleic acid metabolism, ascorbate and aldarate metabolism, and drug metabolism–cytochrome P450 (Fig. 3D), consistent with a metabolism-dominant RNA modification signature. In contrast, Cluster_2 showed enrichment of immune-related and oncogenic signaling pathways, including B-cell receptor and T-cell receptor signaling, ECM-receptor interaction and cell adhesion pathways.

To further characterize the immune contexture associated with these RNA modification patterns, we quantified immune cell infiltration using ssGSEA. Cluster_1 exhibited elevated infiltration of natural killer cells, central memory CD4^+^ and CD8^+^ T cells, effector memory CD4^+^ T cells, and activated CD4^+^ T cells. In contrast, Cluster_2 was characterized by increased abundance of effector memory CD8^+^ T cells, macrophages, and activated B cells (Fig. 3E). Collectively, these findings indicate that distinct RNA modification patterns are closely associated with specific immune landscapes and may influence clinical outcome by reshaping the TME.

### Construction and validation of the RMP_Score scoring system

To quantify the inter-patient heterogeneity in RNA modification patterns, we developed a prognostic scoring system, designated the RMP_Score, derived from DEGs between RNA modification clusters (Cluster_1 and Cluster_2). The risk score was calculated using the following formula:

$$\begin{aligned}\mathrm{RMP}\_\mathrm{Score}=&\left(0.06413236\times\mathrm{expr}\,(\mathrm{CES}1)\right)\\&+\left(0.11206231\times\mathrm{expr}\,(\mathrm{TM}4\mathrm{SF}1)\right)\end{aligned}$$

**Fig. 4:**
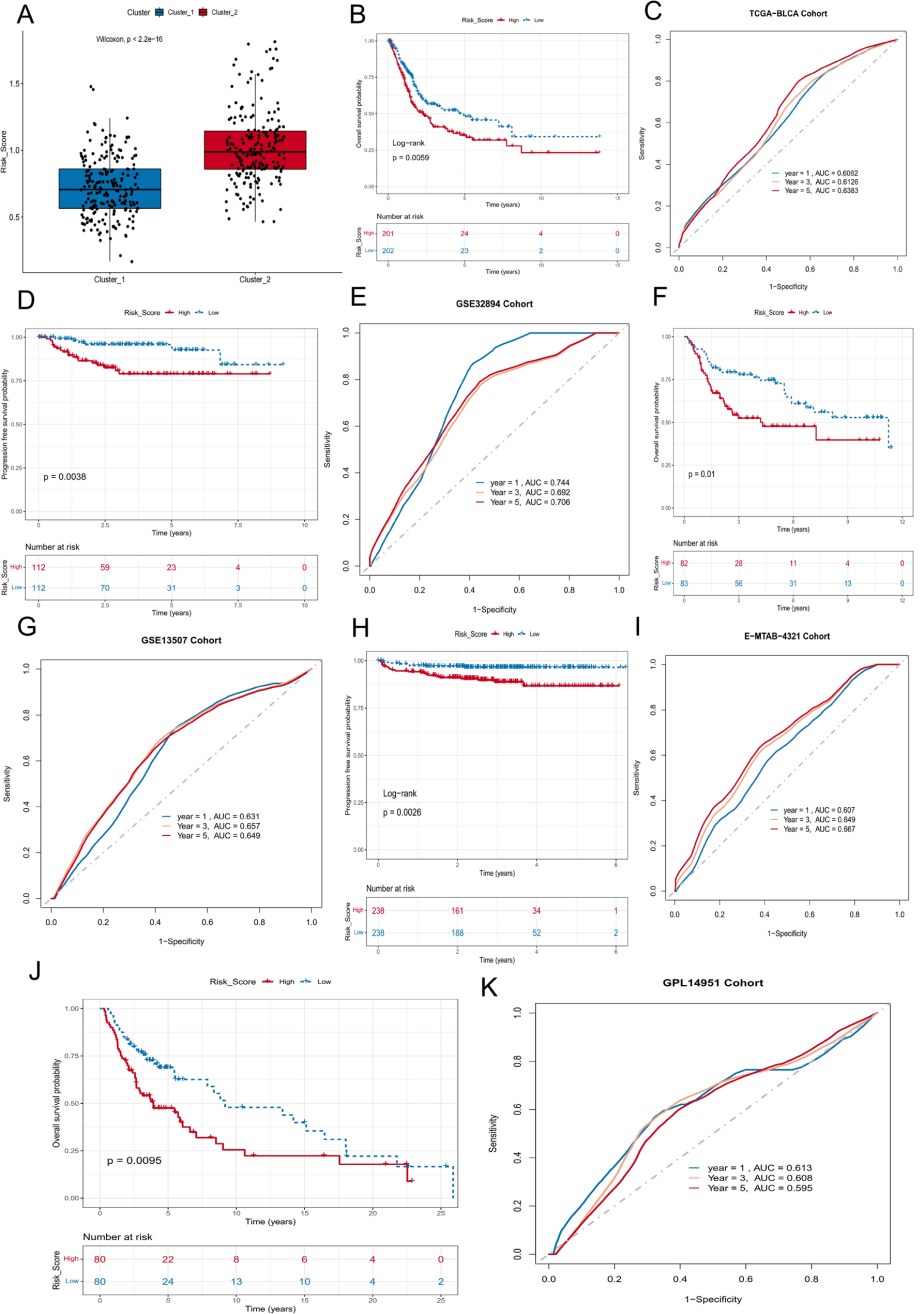
Construction and validation of the RNA modification-based scoring model. **A** Distribution of RMP_Score in two RNA modification clusters, showing significantly lower scores in Cluster_1 compared to Cluster_2. **B** Kaplan–Meier overall survival curves for patients with high versus low RMP_Score in TCGA-BLCA cohort. **C** Prognostic performance of RMP_Score in TCGA-BLCA cohort. **D**–**K** External validation of RMP_Score’s prognostic value in independent cohorts. Kaplan–Meier curves for high- versus low-score groups in GSE32894 (**D**), GSE13507 (**F**), E-MTAB-4321 (**H**), and GSE48276/GSE70691/GSE69795 (**J**). ROC curves for the scoring model’s predictive performance in GSE32894 (**E**), GSE13507 (**G**), E-MTAB-4321 (**I**), and GSE48276/GSE70691/GSE69795 (**K**)

As expected, the RMP_Score was significantly lower in Cluster_1 compared with Cluster_2 (Wilcoxon *P* < 2.2 × 10^− 16^; Fig. 4A), consistent with the better prognosis observed in Cluster_1. Patients in the TCGA-BLCA cohort were subsequently stratified into high- and low-score groups using an optimal cutoff, with the low-score group showing significantly better OS (log-rank *P* = 0.0059; Fig. 4B). Time-dependent ROC analysis yielded area under the curve (AUC) values of 0.61, 0.61, and 0.64 for 1-, 3-, and 5-year OS, respectively (Fig. 4C; Table S4), demonstrating moderate predictive performance.

The robustness of the RMP_Score was further validated in six independent cohorts (GSE48276, GSE70691, GSE69795, GSE13507, E-MTAB-4321, and GSE32894). In all validation datasets, patients with low RMP_Score consistently exhibited better survival (Fig. 4D, F, H, J), and the predictive accuracy was comparable to that observed in the training cohort (Fig. 4E, G, I, K; Table S5), highlighting the stability and reproducibility of the RMP_Score across diverse platforms and patient populations.

### Clinical, chemotherapy, and immunotherapy relevance of the RMP_Score

**Fig. 5:**
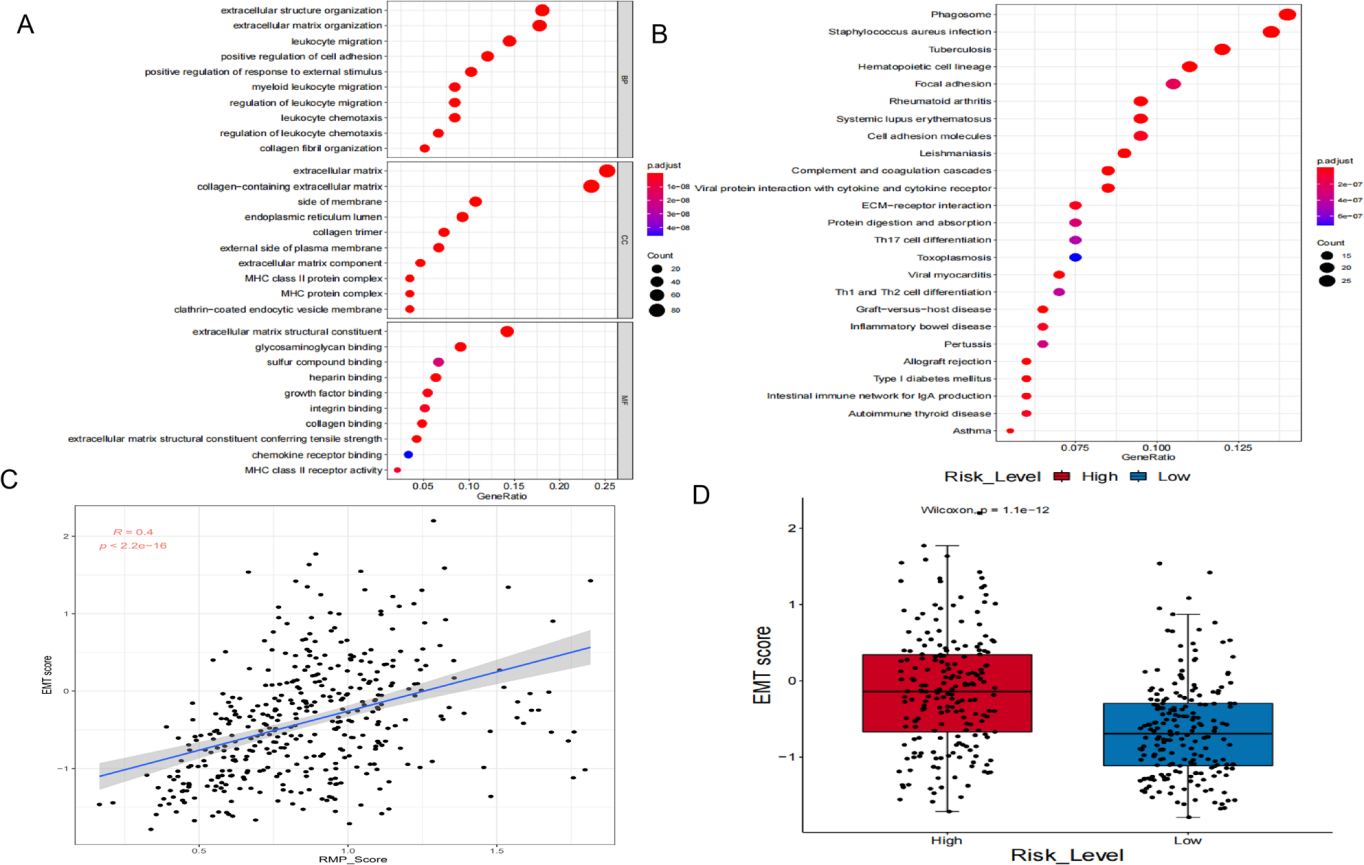
Functional enrichment of differentially expressed genes and association between EMT and RMP_Score. **A**, **B** Gene Ontology (GO) (**A**) and KEGG pathway (**B**) enrichment analysis of differentially expressed genes between high- and low-RMP_Score groups. The x-axis represents the gene ratio for each term, with color intensity indicating enrichment significance. **C** Spearman correlation between EMT scores and RMP_Score in the TCGA-BLCA cohort. **D** Comparison of EMT scores between high (red) and low (blue) RMP_Score groups in the TCGA-BLCA cohort. EMT, epithelial-mesenchymal transition

To further investigate the biological significance of RNA modification patterns, we performed functional enrichment analysis of RNA phenotype-related DEGs. These genes were significantly enriched for processes related to ECM organization and immune cell migration (Fig. 5A). At the pathway level, they were predominantly associated with ECM-receptor interactions and immune-related signaling pathways (Fig. 5B). Given the established link between ECM remodeling and epithelial-mesenchymal transition (EMT), we calculated EMT scores based on the expression levels of 25 epithelial marker genes and 52 mesenchymal marker genes [55]. We found a significant positive correlation between the RMP_Score and EMT scores (*Rs* = 0.4, *P* = 2.2 × 10^− 16^; Fig. 5C). Tumors in the high RMP_Score group exhibited significantly higher EMT scores than those in the low-score group (*P* = 1.1 × 10^− 12^; Fig. 5D), indicating that a high RMP_Score is associated with a more mesenchymal, aggressive tumor phenotype.

**Fig. 6:**
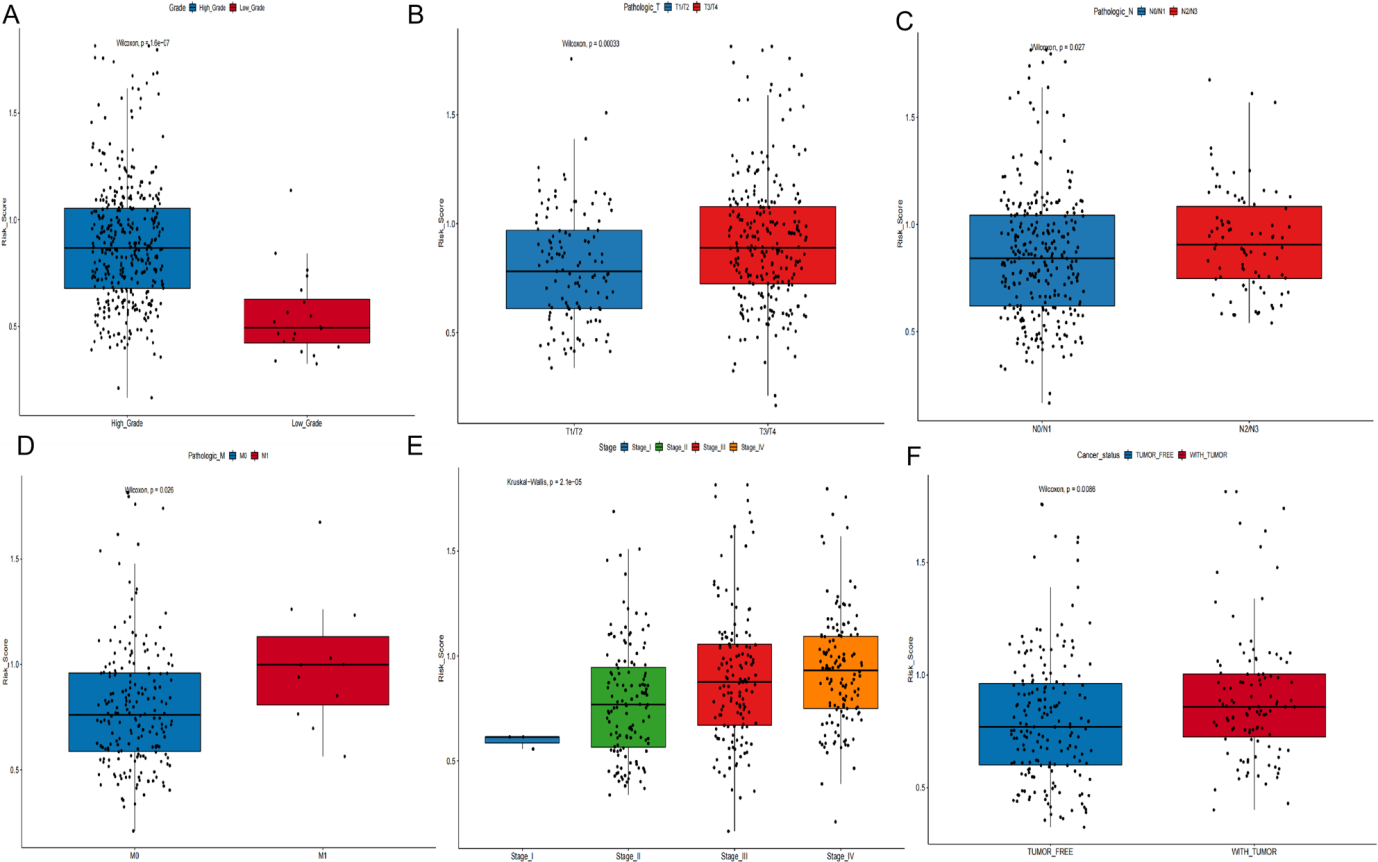
Association between RMP_Score and clinicopathological features in bladder cancer. **A**–**E** Distribution of RMP_Score across tumor grade (**A**) and TNM stages (**B**–**E**) in the TCGA-BLCA cohort. **F** Comparison of RMP_Score between tumor-free (blue) and tumor-bearing (red) groups in the TCGA-BLCA cohort

We next examined the relationship between RMP_Score and clinicopathological characteristics. Elevated RMP_Score values were observed in “high pathological grade and advanced tumor stage (*P* < 0.05; Fig. 6A-E). The RMP_Score was also significantly elevated in patients with residual tumors compared with those without tumors (*P* = 0.0086; Fig. 6F), suggesting its potential as a marker of tumor progression.

**Fig. 7:**
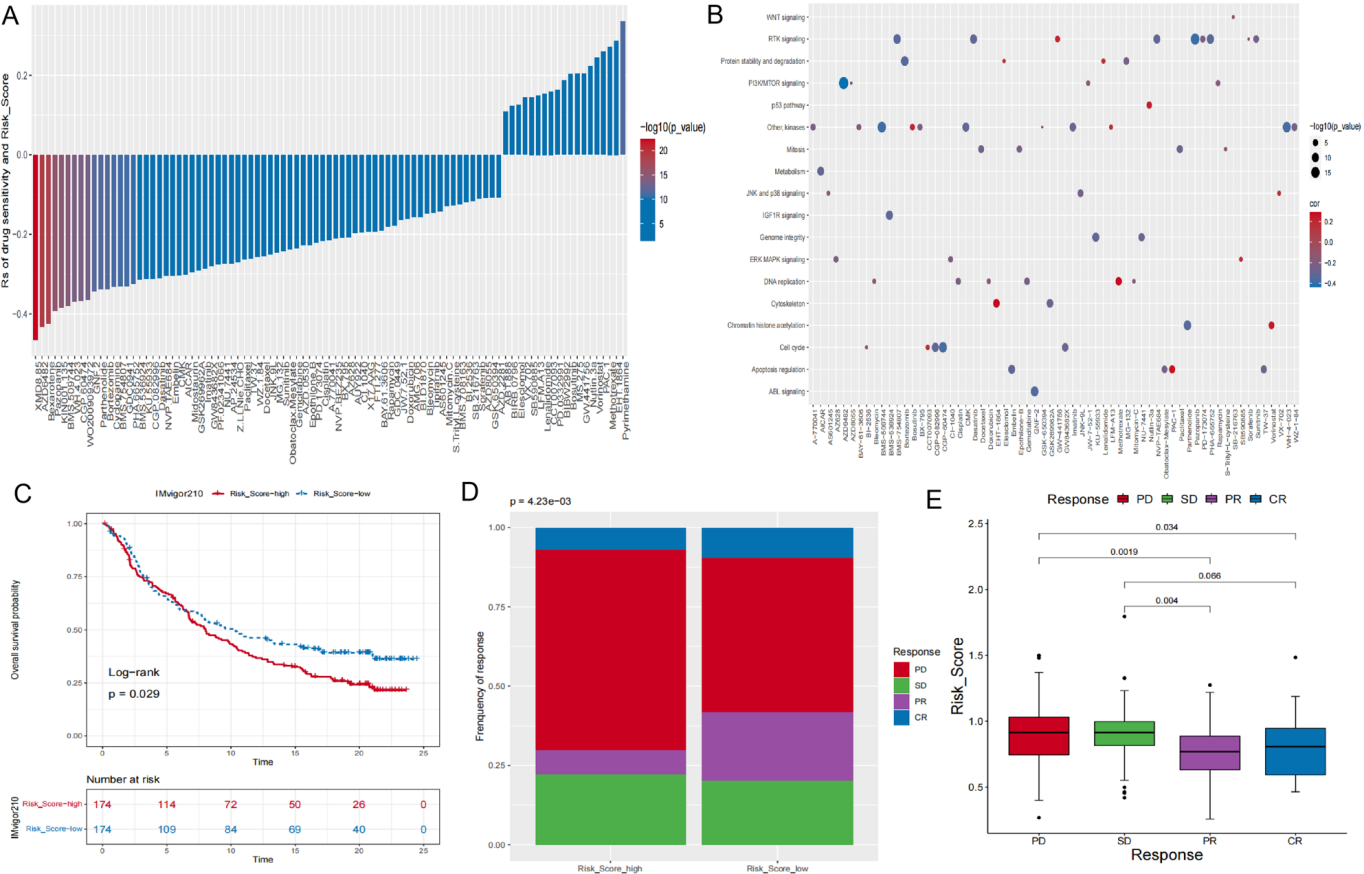
Predictive value of the RMP_Score for chemotherapy and immunotherapy. **A** Spearman correlations between RMP_Score and drug sensitivity. Each bar represents a drug; bar height indicates correlation coefficient (*Rs*), and color intensity reflects statistical significance. *Rs* > 0 indicates relative drug resistance, and *Rs* < 0 indicates relative drug sensitivity. **B** Bubble plot summarizing drugs associated with resistance (red) or sensitivity (blue) in relation to RMP_Score and their targeted signaling pathways. The x-axis shows drugs, the y-axis shows pathways, and bubble size reflects the significance of the correlation. **C** Kaplan-Meier overall survival curves for high- versus low-RMP_Score groups in patients receiving PD-L1 blockade (anti-PD-L1) therapy in the IMvigor210 cohort. **D** Proportions of patients with different clinical responses (CR, PR, SD, PD) to PD-L1 blockade in the IMvigor210 cohort. Statistical significance was assessed using Fisher’s exact test. **E** Distribution of RMP_Score among patients with different clinical outcomes after anti-PD-L1 therapy in the IMvigor210 cohort. CR, complete response; PR, partial response; SD, stable disease; PD, progressive disease

To evaluate the clinical relevance of RMP_Score in chemotherapy response, we correlated RMP_Score with predicted IC50 values for 138 drugs in the TCGA-BLCA cohort. Spearman correlation analysis identified 64 significant associations between the RMP_Score and drug sensitivity (Fig. 7A; Table S6). Among these, 51 drugs were predicted to exhibit enhanced efficacy in high-score tumors, including TW-37 (Bcl-2 family inhibitor; *R*s = − 0.29, *P* = 1.06 × 10^− 7^), PD-173,074 (FGFR inhibitor; *Rs* = − 0.22, *P* = 7.39 × 10^− 6^), and MG-132 (proteasome inhibitor; *Rs* = − 0.25, *P* = 5.78 × 10^− 7^). Thirteen drugs were predicted to exhibit reduced efficacy in high-score tumors, such as EHT-1864 (Rac GTPase inhibitor; *Rs* = 0.29, *P* = 4.46 × 10^− 9^) and GW-441,756 (TrkA inhibitor; *Rs* = 0.20, *P* = 3.60 × 10^− 5^). Pathway enrichment of drug targets showed that drugs predicted to be more effective in high-score tumors predominantly targeted JNK/p38, PI3K/mTOR, and WNT signaling, whereas less effective drugs predominantly targeted apoptotic pathways (Fig. 7B). These results highlight the potential of RMP_Score to guide chemotherapy selection.

We further evaluated the predictive value of RMP_Score for immunotherapy in the IMvigor210 cohort, which includes patients treated with anti-PD-L1 therapy. Patients in the low-score group had significantly better OS following immunotherapy (log-rank *P* = 0.029; Fig. 7C). Among 348 patients, a higher proportion of low-score patients achieved a complete response (CR) or partial response (PR), while stable disease (SD) and progressive disease (PD) predominated in the high-score group (*P* = 4.23 × 10^− 3^; Fig. 7D). Consistently, patients with CR/PR had significantly lower RMP_Score values than those with SD/PD (Fig. 7E). These findings suggest that RMP_Score is a promising biomarker for predicting response to ICB therapy.

### Expression characteristics and functional role of CES1 in bladder cancer

**Fig. 8:**
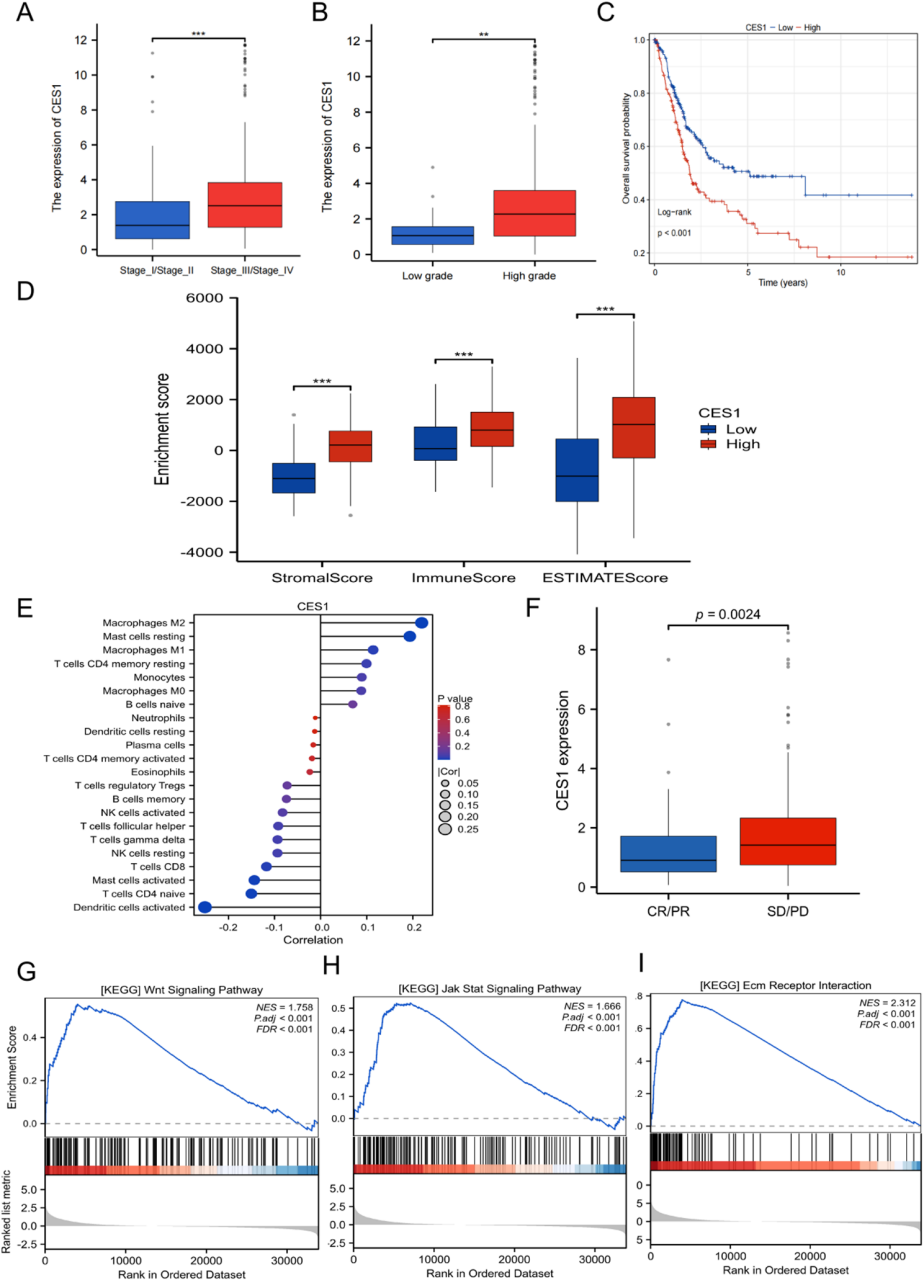
Association of CES1 expression with clinical features, immunotherapy response, and functional enrichment in bladder cancer. **A**, **B** Association of CES1 expression with pathological stage (**A**) and tumor grade (**B**). **C** Kaplan-Meier overall survival curves stratified by CES1 expression. **D**, **E** Relationships between CES1 expression and tumor microenvironment: ESTIMATE scores (**D**) and immune cell fractions inferred by the CIBERSORT algorithm (**E**). **F** Differences in CES1 expression among patients with different responses to immunotherapy. **G**–**I** GSEA of pathways associated with high versus low CES1 expression. GSEA, Gene set enrichment analysis

Among the genes comprising the RMP_Score model, we focused on TM4SF1 and CES1 as potential key regulators. Although TM4SF1 has been relatively well characterized in bladder cancer [56, 57], the role of CES1 remains largely unexplored. In the TCGA-BLCA cohort, high CES1 expression was significantly associated with more advanced pathological stage, higher tumor grade, and poorer prognosis (Fig. 8A-C). Univariable Cox regression analysis including CES1 expression, tumor stage, and tumor grade showed that higher CES1 expression was associated with an increased hazard (HR = 1.676, 95% CI 1.244–2.259; *P* < 0.001). After adjustment for stage and grade in the multivariable Cox model, CES1 remained an independent prognostic factor (HR = 1.446, 95% CI 1.065–1.964; *P* = 0.018; Supplementary Figure S1). Consistent with the transcriptomic findings, analysis of IHC data from the HPA showed that CES1 protein staining was more intense and widespread in high-grade urothelial carcinomas than in low-grade tumors (Supplementary Figure S2). These results indicate that the association between CES1 expression and tumor aggressiveness extends to the protein level and is not restricted to mRNA-based measurements.

To investigate the potential impact of CES1 on the TIME, we applied both ESTIMATE [58] and CIBERSORT [59] algorithms. Comparison of high- and low-CES1 expression groups revealed significantly higher stromal, immune, and ESTIMATE scores in the high-CES1 group (*P* < 0.001; Fig. 8D), suggesting increased abundance of stromal and immune components in these tumors.

Further immune-cell deconvolution via CIBERSORT revealed distinct associations between CES1 expression and immune cell subsets. CES1 expression positively correlated with the infiltration of M2 macrophages (*Rs* = 0.219, *P* < 0.001) and resting mast cells (*Rs* = 0.194, *P* < 0.001), respectively (Fig. 8E). In contrast, negative correlations were observed with activated dendritic cells (*Rs* = − 0.251, *P* < 0.001) and naive CD4^+^ T cells (*Rs* = − 0.150, *P* < 0.01), respectively (Fig. 8E). These findings suggest that elevated CES1 expression is associated with an immunosuppressive microenvironment characterized by pro-tumorigenic immune populations yet attenuated anti-tumor immunity. In the IMvigor210 cohort, CES1 expression was significantly lower in patients who achieved CR/PR than those with SD/PD (*P* = 0.0024; Fig. 8F), further supporting its role in tumor progression and resistance to immunotherapy. In the TCGA-BLCA cohort, Spearman correlation analysis between CES1 and immune checkpoint genes showed that CES1 expression was positively correlated with correlation coefficients greater than 0.2 and P values < 0.001 (Supplementary Figure S3). By contrast, the association between CES1 and CD274 (PD-L1) was weak and did not reach statistical significance (Rs = 0.064, *P* = 0.195; Supplementary Figure S3). These findings suggest that CES1 expression is more closely associated with a broader immune exhaustion program than with CD274 transcription alone. To explore the biological pathways associated with CES1, we performed Gene Set Enrichment Analysis (GSEA). Tumors with high CES1 expression showed significant enrichment in several key signaling pathways, notably the WNT, JAK-STAT, and ECM-receptor interaction pathways (Normalized Enrichment Score > 1.60, False Discovery Rate < 0.001; Fig. 8G–I). These results suggest that CES1 may play a functional role in regulating processes critical for tumor progression, immune modulation, and remodeling of the TME.

**Fig. 9:**
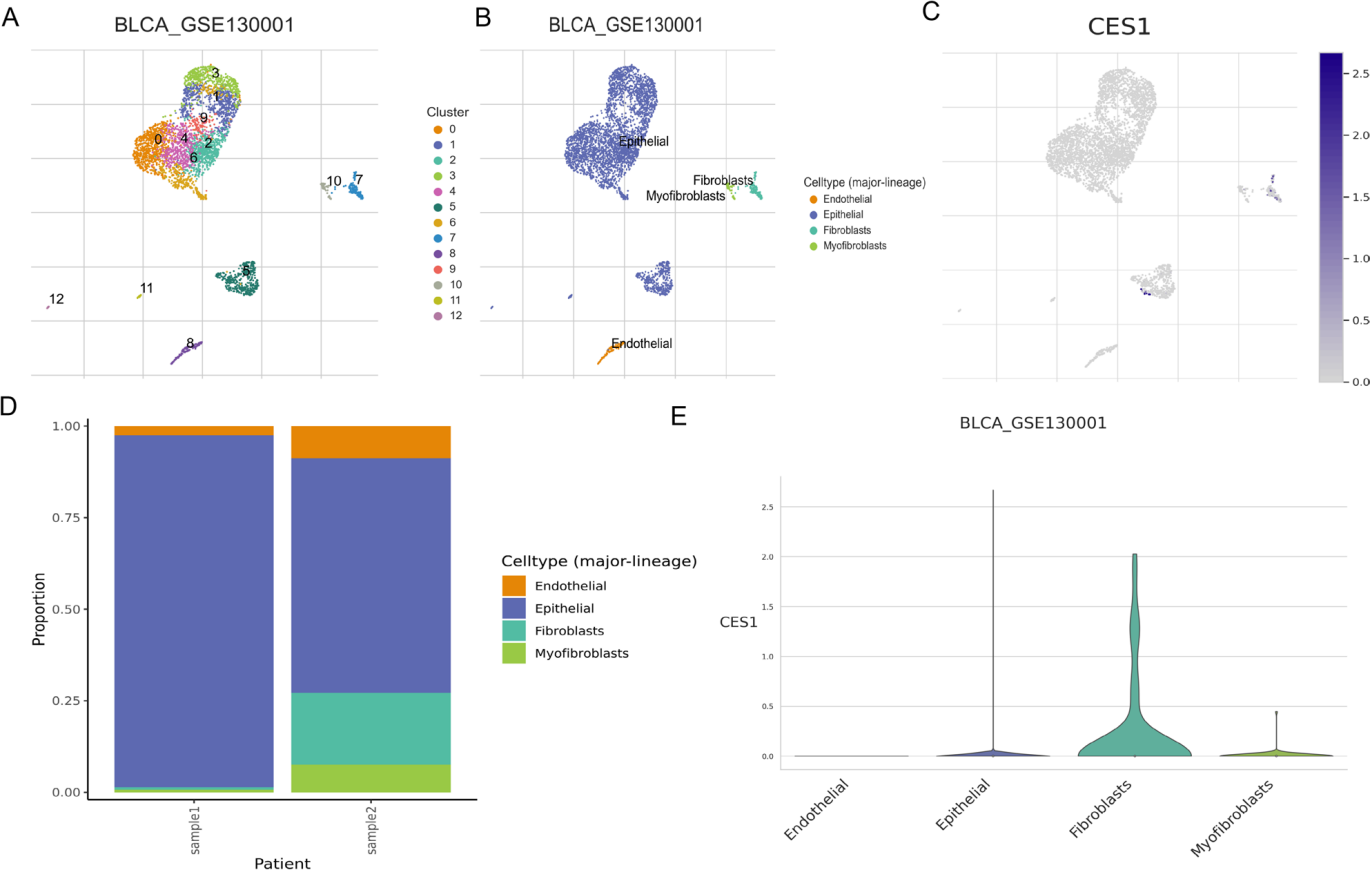
Single-cell transcriptomic analysis of CD45-negative bladder cancer cells reveals cell-type heterogeneity and CES1 expression patterns. **A** UMAP plot showing unsupervised clustering of CD45-negative cells from bladder cancer samples (GSE130001), identifying 13 transcriptionally distinct clusters (0–12). **B** Annotation of major clusters into four cell types: epithelial cells, fibroblasts, myofibroblasts, and endothelial cells. **C** UMAP feature plot displaying CES1 expression across single cells. **D** Stacked bar chart comparing the proportions of major cell types between two bladder cancer samples (“sample1” and “sample2”). **E** Violin plots depicting CES1 expression levels among the four annotated cell types. UMAP, Uniform Manifold Approximation and Projection

To further dissect the cellular heterogeneity of the bladder cancer microenvironment, we performed unsupervised clustering of CD45^−^ cells from the GSE130001 dataset. Uniform Manifold Approximation and Projection (UMAP) visualization identified 13 transcriptionally distinct cell clusters, highlighting the complexity of non-immune cellular constituents within the TME (Fig. 9A). These clusters were annotated into four major lineages: epithelial cells (comprising both tumor and normal epithelium), fibroblasts, myofibroblasts (activated stromal cells), and endothelial cells (Fig. 9B). Analysis of CES1 expression across these clusters revealed detectable expression in epithelial cells, fibroblasts, and myofibroblasts, with particularly high expression in fibroblast-enriched regions (Fig. 9C). Comparative analysis between two representative samples demonstrated a pronounced expansion of fibroblast and myofibroblast populations in sample 2 (Fig. 9D). Violin plot quantification confirmed that CES1 expression was enriched in fibroblasts, with lower levels in myofibroblasts and epithelial cells (Fig. 9E), indicating a prominent role of CES1 in the stromal compartment of the bladder cancer TME.

**Fig. 10:**
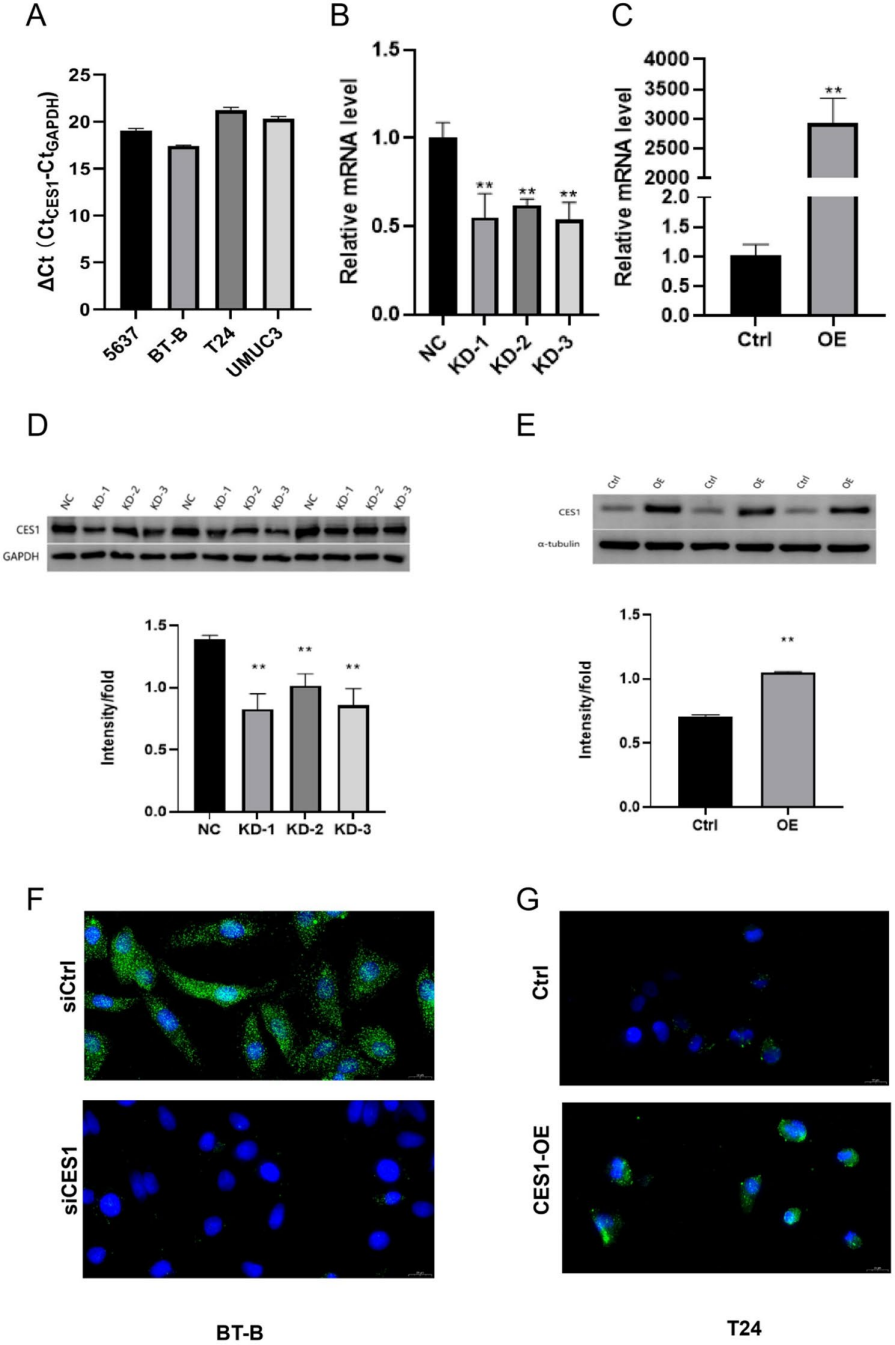
Expression characteristics of CES1 in bladder cancer cell lines. **A** qPCR analysis of baseline CES1 mRNA levels in bladder cancer cell lines T24, 5637, UMUC3, and BT-B. **B**, **C** qPCR validation of CES1 knockdown (**B**) and overexpression (**C**) efficiency. **D**, **E** Western blot confirmation of CES1 protein knockdown (**D**) and overexpression (**E**). **F**, **G** Immunofluorescence staining showing CES1 expression after knockdown (**F**) and overexpression (**G**). qPCR, quantitative PCR

We next examined CES1 expression across bladder cancer cell lines. qPCR analysis revealed the highest expression of CES1 in BT-B cells and the lowest in T24 cells (Fig. 10A). Based on these endogenous expression patterns, we performed CES1 knockdown in BT-B cells and ectopic overexpression in T24 cells. Western blotting, qPCR, and immunofluorescence analyses confirmed efficient CES1 silencing in BT-B cells and robust overexpression in T24 cells transfected with pCDH-CMV-CES1 (Fig. 10B-G; Table S7).

**Fig. 11:**
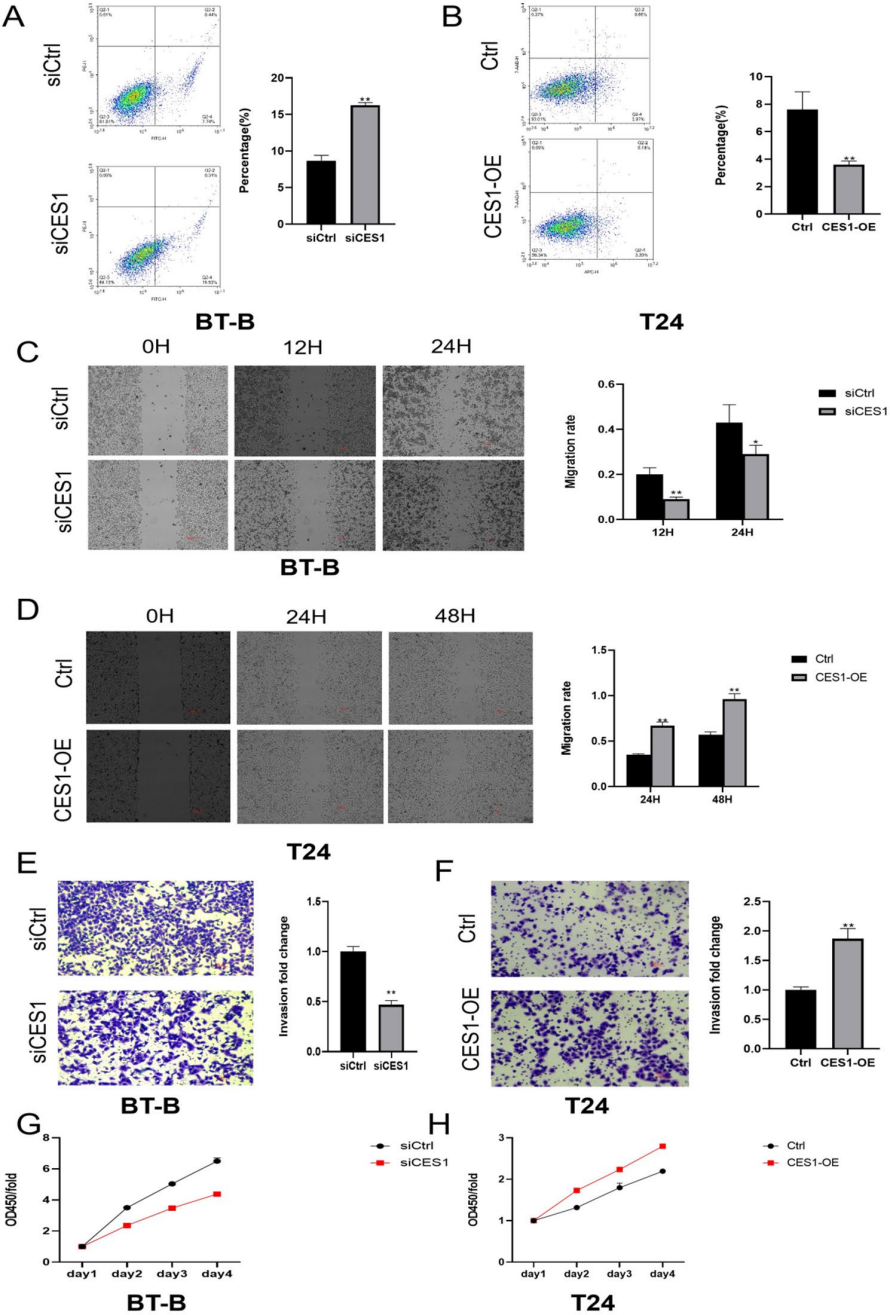
Functional role of CES1 in bladder cancer cells. **A**, **B** Apoptosis analysis after CES1 knockdown (**A**) or overexpression (**B**) by PI/Annexin V double-staining flow cytometry. **C**, **D** Wound-healing assays assessing cell migration after CES1 knockdown (**C**) or overexpression (**D**). **E**, **F** Transwell invasion assays (+ ECM) evaluating invasive capacity after CES1 knockdown (**E**) or overexpression (**F**). **G**, **H** CCK-8 assays measuring cell proliferation after CES1 knockdown (**G**) or overexpression (**H**). ^*^P < 0.05, ^**^P < 0.01. ECM, extracellular matrix

Functionally, CES1 knockdown in BT-B cells significantly induced apoptosis, while CES1 overexpression in T24 cells conferred apoptosis resistance (Fig. 11A, B). In wound-healing assays, CES1 silencing attenuated migratory capacity in BT-B cells, whereas CES1 overexpression enhanced migration in T24 cells (Fig. 11C, D). Transwell assays demonstrated reduced invasive potential after CES1 knockdown and increased invasiveness after CES1 overexpression (Fig. 11E, F). Consistent with these results, CCK-8 proliferation assays revealed that CES1 silencing suppressed proliferation in BT-B cells, whereas CES1 overexpression promoted proliferation in T24 cells (Fig. 11G, H). Collectively, these findings establish a pro-oncogenic role for CES1 in bladder cancer, characterized by enhanced proliferation, migration, and invasion, concomitant with suppression of apoptotic cell death.

## Discussion

To integrate RNA modifications with established bladder cancer molecular subtypes, these epitranscriptomic marks can be conceptualized as a post-transcriptional regulatory layer that amplifies oncogenic signaling outputs on which distinct tumor subtypes depend. The current consensus classification defines distinct expression classes, including luminal papillary, basal squamous, and stroma-rich tumors, which differ in dominant signaling programs and immune-stromal contexts [60, 61]. m^6^A is well positioned to shape these states because it can change RNA stability and translation efficiency in a target selective manner [62]. In bladder cancer models, METTL3-mediated m^6^A modification has been reported to support an AFF4-NF-kB-MYC regulatory network, providing a concrete example of how m^6^A can reinforce proliferative and inflammatory transcriptional outputs that vary across subtypes [63]. A related mechanism links stress signaling to immune programs, JNK and c-Jun upregulate METTL3 and increase m^6^A deposition on PD-L1 mRNA, thereby stabilizing PD-L1 and reducing CD8^+^ T cell-mediated cytotoxicity, which may contribute to immune dysfunction even in the presence of immune infiltration [64]. m^6^A may also intersect with metabolic stress responses. WTAP has been reported to install m^6^A on nuclear factor erythroid 2-related factor 2 (NRF2) mRNA and enhance its stability, thereby supporting ferroptosis resistance, a feature that could be relevant in stress-adapted tumor subtypes [65]. m^1^A adds another regulatory layer. TRMT6-TRMT61A-dependent m^1^A modification of tRNA-derived fragments has been linked to altered gene-silencing activity and regulation of the unfolded protein response in urothelial carcinoma, and depletion of TRMT6/TRMT61A reduces proliferation and cellular stress tolerance in bladder cancer models [66, 67]. Together, these findings support a framework in which m^6^A and m^1^A modifications help maintain subtype-specific transcriptional identities by selectively stabilizing RNAs that connect lineage regulators and key signaling pathways to the final expression state.

Taken together, emerging evidence suggest that RNA modification-associated metabolic remodeling does not merely accompany bladder cancer progression but also contributes to shaping the local immune microenvironment and may influence tumors responses to immunotherapy [68–70]. Recent studies in other solid tumors have shown that disulfidptosis-related gene programs and machine-learning-defined TIME subtypes capture coordinated changes in metabolism and immunity, enabling stratification of patients by prognosis and sensitivity to ICB [71, 72]. In parallel, comprehensive analyses of metabolic reprogramming within the TIME highlight how altered utilization of metabolic pathways create both barriers and therapeutic opportunities for restoring antitumor immunity [73].

In this study, we integrated several independent cohorts to profile four classes of adenosine-related RNA modification writer genes in bladder cancer, encompassing genomic alterations, copy-number variations, and transcriptional dysregulation. Based on these analyses, we derived an RMP_Score and identified CES1 as a functionally relevant gene associated with tumor progression. These findings link RNA modification patterns with molecular subtype heterogeneity, microenvironmental remodeling, and variability in immunotherapy outcomes. The overall modeling strategy aligns with recent multi-omics and machine-learning frameworks developed for prognostic stratification and treatment guidance in urothelial cancer [48–52]. Although these models differ in their specific input layers and clinical endpoints, they collectively support the concept that clinically informative scores can be derived from high-dimensional molecular features.

Our genomic analyses revealed that RNA modification writer genes frequently undergo somatic mutations and CNVs in bladder cancer, accompanied by widespread transcriptional dysregulation. Among these genes, METTL3, KIAA1429, PCF11, and ZC3H13 exhibited relatively high mutation frequencies, and mutations in any of these genes were significantly associated with inferior OS. These alterations likely disrupt epitranscriptomic homeostasis, thereby promoting tumor initiation and progression. Additionally, copy-number gains of ADAR, ADARB2, CLP1, and KIAA1429 were strongly correlated with their overexpression, highlighting gene amplification as a key mechanism driving transcriptional activation. Although the individual roles of m^6^A, m^1^A, APA, and A-to-I editing in cancer biology have been increasingly recognized, integrated analyses of multiple “writer” gene families in bladder cancer remain limited. Our multilayered approach, spanning somatic mutations, CNV, and transcriptomic alterations, provides a more comprehensive framework for understanding how RNA modification networks are dysregulated in this malignancy.

Using unsupervised consensus clustering based on the expression of 30 RNA modification writer genes, we identified two distinct epitranscriptomic subtypes. Cluster_1 was characterized by enrichment of metabolic pathways, including lipid and drug metabolism, and was associated with favorable survival outcomes. In contrast, Cluster_2 exhibited enrichment of immune- and stromal-related pathways, such as immune receptor signaling and ECM-receptor interaction. Despite immune pathway activation, Cluster_2 demonstrated inferior prognosis. This paradoxical finding may be attributed to elevated regulatory T cell infiltration, which fosters an immunosuppressive microenvironment and attenuates effective anti-tumor immunity [74]. Importantly, closer examination of the immune composition of Cluster_2 indicates that the observed increase in immune cells was not primarily driven by cytotoxic effectors. Instead, the analysis revealed a shift toward suppressive populations, including higher levels of myeloid-derived suppressor cells (MDSCs) and tumor-associated macrophages (TAMs), together with increased neutrophils and mast cells. This pattern is consistent with an immune infiltrate that is abundant but functionally restrained, in which immune cells are present yet organized in ways that limit effective tumor killing. Mechanistically, MDSCs can blunt T-cell activation through metabolic depletion and inhibitory mediators, contributing to immune escape and treatment resistance in bladder cancer [75]. TAMs, especially those exhibiting an M2-like program, can reinforce immunosuppression via IL-10 and TGF-β signaling, reduce antigen presentation, and promote stromal remodeling that impedes productive T cell engagement [76]. Mast cells may further support immunosuppression by promoting angiogenesis, tissue remodeling, and immunomodulatory signaling in urothelial carcinoma [77].

In this context, neutrophil enrichment may not simply reflect inflammation: Neutrophil extracellular traps (NETs) have been implicated in bladder cancer radioresistance and can contribute to an immunosuppressive microenvironment [78]. More recently, a positive feedback mechanism involving NETs and stanniocalcin 1 (STC1) has been reported to promote immune evasion and immunotherapy resistance, providing a mechanistic link between neutrophil-dominant inflammation and poor checkpoint blockade response [79]. Collectively, these observations support the view that Cluster_2 represents a myeloid-dominant, stroma-supported immunosuppressive state, which may help underlie its poorer prognosis and reduced responsiveness to immunotherapy.

To assess RNA modification status at the individual patient level, we developed the RMP_Score, derived from DEGs distinguishing Cluster_1 and Cluster_2. The RMP_Score effectively stratified patients according to survival outcomes in the TCGA-BLCA cohort, and its prognostic value was further validated in six independent datasets. CES1 demonstrated independent prognostic value in multivariable models (HR = 1.446, 95% CI = 1.065–1.964; *P* = 0.018; Supplementary Figure S1). In addition, the RMP_Score was associated with therapeutic responses. Drug sensitivity analyses indicated that tumors with high scores were more likely to respond to agents targeting the JNK/p38, PI3K/mTOR, and WNT signaling pathways, while showing resistance to drugs that target apoptotic processes. One possible interpretation is that high score tumors occupy a stress-adapted, signaling-dependent state, relying on stress kinases, growth pathways, and lineage- or stemness-associated programs, which may create actionable dependencies. Concurrently, reduced apoptotic priming and compensatory anti-apoptotic BCL-2 family activity could contribute to diminished responses to apoptosis -targeting agents [80, 81].

In the context of immunotherapy, patients with low RMP_Score in the IMvigor210 anti-PD-L1 cohort demonstrated superior OS and higher objective response rates. These findings suggest that distinct RNA modification landscapes may influence antigen presentation, immune checkpoint expression, and the abundance and functional states of infiltrating immune cells, thereby contributing to heterogeneous clinical responses to ICB.

From a clinical perspective, the RMP_Score may be valuable because it condenses complex RNA-modification-related programs into a single readout that captures both tumor-intrinsic biology and the immune-stromal context. Being expression-based, the score could, in principle be measured from routine transurethral resection of bladder tumor (TURBT) or biopsy specimens using a focused RNA panel on formalin-fixed, paraffin-embedded tissue, consistent with ongoing efforts to translate transcriptomic classifiers into more practical tools for bladder cancer stratification [60]. In clinical workflows, one near-term application would be treatment selection for ICB. In IMvigor210, patients with lower RMP_Score exhibited better outcomes under anti-PD-L1 therapy, consistent with prior analyses showing that response is associated with a pre-existing CD8 T-effector phenotype and higher tumor mutation or neoantigen burden, whereas stromal programs such as TGF-β signaling correlate with T-cell exclusion and reduced benefit from PD-L1 blockade [53, 82]. A second application is to complement actionable genomics in selecting targeted therapies or trials. FGFR2/3-altered urothelial cancers constitute a defined subgroup with established sensitivity to FGFR inhibition, with erdafitinib demonstrating meaningful activity in early studies and an OS advantage versus chemotherapy in a randomized phase Ⅲ trial [83, 84]. Collectively, these data support a practical model in which the RMP_Score is interpreted alongside standard clinicopathologic variables and key molecular alterations to guide prioritization of PD-(L)1 monotherapy versus combination strategies and referral to targeted or biomarker-driven trials, while prospective validation remains necessary prior to clinical implementation.

In the RMP_Score model, we identified CES1 as a novel and robust prognostic biomarker, closely associated with poor clinical outcomes and resistance to ICB in bladder cancer. Elevated CES1 expression was significantly associated with advanced tumor stage, high pathological grade, and elevated stromal and immune scores. CES1 expression was associated with the establishment of an immunosuppressive TME, characterized by an increased infiltration of M2 macrophages and resting mast cells, concomitant with reduced infiltration of cytotoxic lymphocytes. CES1, a member of the mammalian serine hydrolase family [85], is primarily expressed in metabolically active tissues such as the liver and adipose tissues [86, 87]. It catalyzes the hydrolysis of lipid esters and thioesters, playing a crucial role in lipid metabolism and systemic energy homeostasis [86]. Consistent with its known biological functions, our functional enrichment analyses indicated that CES1 expression was positively associated with WNT signaling, JAK-STAT signaling, and ECM-receptor interactions. Collectively, these findings suggest that CES1 functions as a key regulator of TME remodeling in bladder cancer, potentially driving immunotherapy resistance through modulation of stromal architecture and immune cell infiltration patterns.

We also examined IHC data from the HPA and observed stronger CES1 staining in high-grade urothelial carcinomas than in low-grade tumors (Supplementary Figure S2). These findings support a link between CES1 expression and tumor aggressiveness that extends beyond mRNA measurements. Given that CES1 family enzymes participate in xenobiotic and drug metabolism [88], elevated CES1 expression may influence treatment outcomes not only by modulating the TME but also by altering intratumoral drug handling in specific therapeutic contexts.

Experimental validation robustly substantiated the clinical relevance of CES1 in bladder cancer. CES1 knockdown significantly attenuated tumor cell proliferation, viability, invasion, and migratory capacity in vitro. ScRNA-seq revealed that CES1 expression was predominantly localized to CAFs within the TME, providing critical cellular context for its mechanistic role. We hypothesize that elevated CES1 expression in CAFs reprograms lipid metabolism to generate energetic and biosynthetic substrates necessary for ECM crosslinking and remodeling, consistent with the observed enrichment of ECM-receptor interaction pathways (Fig. 8I). High CES1-expressing CAF states are accompanied by metabolic reprogramming that supports ECM production and crosslinking and generates lipid-derived signals that sustain CAF activation. These signals may also influence tumor cells via paracrine communication, consistent with EMT-like features and increased invasiveness. CES1 may act in a cell type-dependent manner, with CAF programs primarily driving matrix remodeling and inflammatory signaling, whereas tumor epithelial programs may be more related to intracellular lipid handling and mitochondrial fitness under stress. These observations align with the broader concept of CAF-tumor metabolic coupling in invasion and therapy resistance [89, 90].

This interpretation is also compatible with reports describing reinforcing links between ECM remodeling and metabolic rewiring during progression and drug resistance [91]. In bladder cancer, ECM stiffening has been linked to WNT activation and stemness [92], which offers a potential bridge between CES1 associated stromal remodeling and WNT pathway engagement. More broadly, ECM stiffness can reshape immune infiltration and resistance through mechanosensing programs, supporting the possibility that CES1 linked stromal remodeling contributes to sustained WNT signaling and cytokine and JAK-STAT activity [93].

Given that CES1 knockdown reduced migration and invasion in our assays, CES1 may be linked to metastatic programs that depend on ECM remodeling and pro migratory signaling, although this requires direct experimental validation. A plausible link is TGF-β-associated EMT and motility [94]. Invasion is shaped not only by biochemical cues but also by the physical and biomechanical tumor microenvironment, which can cooperate with signaling programs to facilitate tissue infiltration [95]. Within this context, matrix metalloproteinases (MMP1 and MMP2) are strong candidates, as they directly support local matrix degradation and invasion. Recent studies have identified MMP1 positive malignant subsets with distinct tumor-immune interactions, suggesting that MMP1-high states align with aggressive behavior in a microenvironment-coupled setting [96]. MMP1 can also be regulated by stress and inflammation pathways such as JNK and c-Jun, providing a feasible route by which pro-migratory signaling converges on MMP1 [97]. For MMP2, transcriptional regulation has been linked to metastasis control [98], and JNK-related signaling has been associated with MMP2 dependent invasion in experimental models [99]. Structurally, metastatic dissemination often involves invadopodia, ECM-degrading protrusions enriched for MMP activity [100]. Finally, metastasis is increasingly viewed as a microenvironment-assisted process in which stromal and immune cues establish permissive conditions beyond the primary tumor [101]. Together, these studies support a testable hypothesis that CES1-high states may track with enhanced TGF-β-driven motility and MMP1/MMP2-associated ECM remodeling.

Our CIBERSORT analysis revealed a positive correlation between CES1 expression and estimated M2 macrophage infiltration. Macrophage polarization is sensitive to lipid availability and fatty acid oxidation programs [102], thus, CES1-driven hydrolysis of lipid esters could reshape the local lipid environment, favoring an M2-like immunosuppressive state. This interpretation is consistent with recent immunometabolism studies highlighting fatty acid oxidation, cholesterol handling, and phospholipid remodeling as key determinants of TAM phenotypes [103], and with bladder cancer studies linking immunometabolic remodeling to resistance to checkpoint blockade [104].

We further examined correlations between CES1 and immune checkpoint markers in the TCGA-BLCA cohort. CES1 expression was positively correlated with PDCD1, TIGIT, BTLA, CTLA4, and HAVCR2, with Spearman correlation coefficients > 0.2 and *P* < 0.05 (Supplementary Figure S3). In contrast, the association with CD274 (PD-L1) was weak and not statistically significant (Spearman *r* = 0.064, *P* = 0.195). Several factors may account for this pattern: CD274 is highly inducible and context-dependent, strongly regulated by interferon-γ and JAK-STAT signaling, and bulk RNA sequencing measurements are influenced by tumor purity and the extent of immune and stromal admixture [105]. In addition, PD-L1 abundance at the cell surface is regulated post-translationally, including via glycosylation, ubiquitination and phosphorylation; thus, mRNA levels do not necessarily reflect functional protein presentation [106]. Overall, these data suggest that CES1-associated immunosuppression may align more closely with broader exhaustion programs than with CD274 transcription alone.

From a therapeutic perspective, it is appealing to consider selective CES1 inhibitors or modulators in combination with pathway-targeted agents, such as PI3K-mTOR or WNT inhibitors, or with ICB. At the same time, on-target toxicity in metabolically active organs and compensation through parallel pathways are realistic concerns that would require careful evaluation in preclinical models and in early-phase clinical studies [107].

This study has several limitations. Most mechanistic inferences derived from correlative analyses of public datasets combined with preliminary in vitro experiments. The precise mechanisms by which CES1 rewires lipid metabolism in CAFs and how these changes influence remodeling and immune regulation remain to be defined. Addressing these questions will require dedicated studies in co-culture systems incorporating CAFs, tumor cells, and macrophages, as well as conditional models and integrated metabolomic and lipidomic profiling. Although CES1 demonstrated independent prognostic value in multivariable analyses (HR = 1.446, 95% CI 1.065–1.964; *P* = 0.018; Supplementary Figure S1), larger, harmonized cohorts with comprehensive pathological and treatment annotations will be essential to refine the estimated effect size and to test generalizability.

## Conclusions

In summary, we systematically profiled four classes of adenosine-related RNA modification writer genes in bladder cancer, revealing frequent genomic and CNVs, and widespread transcriptional dysregulation. These molecular perturbations defined distinct RNA modification signatures, each associated with unique TME compositions and divergent clinical outcomes.

Based on these patterns, we developed the RMP_Score, which effectively stratified patient risk across multiple independent cohorts. The RMP_Score further demonstrated predictive value for differential sensitivity to targeted therapies and cytotoxic agents, as well as clinical responses to anti-PD-L1 immunotherapy. These findings establish the RMP_Score as a promising tool for risk stratification and therapeutic decision-making in bladder cancer. Furthermore, we identified CES1 as a critical determinant within this scoring system, with elevated expression predicting adverse prognosis and resistance to ICB. Through integrated analysis of bulk and single-cell transcriptomic data, complemented by functional experiments, we demonstrated that CES1 drives tumor progression via lipid metabolic reprogramming in both bladder cancer cells and CAFs. This metabolic adaptation facilitates ECM remodeling and fosters an immunosuppressive TME, contributing to therapeutic resistance.

This study underscores the pivotal role of RNA modification networks, particularly the writer gene CES1, in bladder cancer pathogenesis. The findings establish a foundation for future investigations into the mechanistic roles of RNA modifications and CES1, and highlight the translational potential of RMP_Score and CES1 as complementary prognostic and predictive biomarkers. Prospective clinical validation and deeper elucidation of CES1 and RMP_Score are anticipated to facilitate their integration into personalized therapeutic strategies for bladder cancer.

## Supplementary Information

Supplementary Material 1.

Supplementary Material 2.

## Data Availability

Publicly available datasets were analyzed in this study. The dataset analyzed for this study is available from the corresponding author upon reasonable request.

## Abbreviations

A-to-I: Adenosine-to-inosine
APA: Alternative polyadenylation
APC: Allophycocyanin
BLCA: Bladder urothelial carcinoma
CAF: Cancer-associated fibroblast
CCK-8: Cell Counting Kit-8
CES1: Carboxylesterase 1
CT: Cycle threshold
DEG: Differentially expressed gene
EMT: Epithelial-mesenchymal transition
GSEA: Gene Set Enrichment Analysis
GSVA: Gene Set Variation Analysis
HALLMARK: Hallmark gene set (Molecular Signatures Database collection)
IC50: Half-maximal inhibitory concentration
ICB: Immune checkpoint blockade
IMvigor210: IMvigor210 clinical trial / cohort
LASSO: Least absolute shrinkage and selection operator
m^1^A: N1-methyladenosine
m^5^C: 5-methylcytidine
m^6^A: N6-methyladenosine
m^7^G: 7-methylguanosine
OS: Overall survival
RMP_Score: RNA modification-based score
ROC: Receiver operating characteristic
AUC: Area under the curve
scRNA-seq: Single-cell RNA sequencing
ssGSEA: Single-sample gene set enrichment analysis
TCGA-BLCA: The Cancer Genome Atlas – Bladder urothelial carcinoma
TIL: Tumor-infiltrating lymphocyte
TIME: Tumor immune microenvironment
TISCH2: Tumor Immune Single-cell Hub 2
TME: Tumor microenvironment
TPM: Transcripts per million
Tregs: Regulatory T cells
TRIzol: Commercial reagent for total RNA isolation
UMAP: Uniform Manifold Approximation and Projection
UTR: Untranslated region
