# Supplementary material for: Integrative analysis of adenosine-related RNA modifications defines molecular subtypes of bladder cancer and identifies CES1 as a driver of tumor progression and immunotherapy resistance
Source: BMC Cancer. 2026 Mar 25;26:562. doi: 10.1186/s12885-026-15897-4 (PMC13137507; doi:10.1186/s12885-026-15897-4)
Supplement: Supplementary file 2 — Supplementary Material 2. [file 12885_2026_15897_MOESM2_ESM.pdf]

A

Univariate analysis

Characteristics	Total(N)	HR(95% CI)	P value
Pathologic stage	409		
Stage I&Stage II	133	Reference	
Stage III&Stage IV	276	2.267 (1.567 - 3.281)	< 0.001
Histologic grade	408		
High grade	387	Reference	
Low grade	21	0.338 (0.084 - 1.365)	0.128
CES1	411		
Low	206	Reference	
High	205	1.676 (1.244 - 2.259)	< 0.001

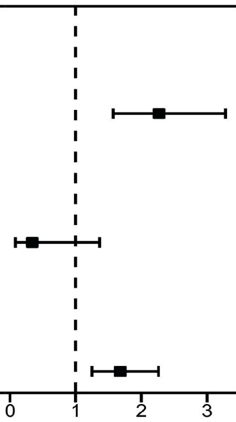

B

Multivariate analysis

Characteristics	Total(N)	HR(95% CI)	P value
Pathologic stage	409		
Stage I&Stage II	133	Reference	
Stage III&Stage IV	276	1.997 (1.364 - 2.923)	< 0.001
Histologic grade	408		
High grade	387	Reference	
Low grade	21	0.567 (0.138 - 2.333)	0.432
CES1	411		
Low	206	Reference	
High	205	1.446 (1.065 - 1.964)	0.018

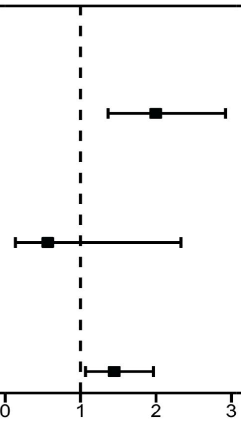

Figure S1 Univariate (A) and Multivariate Cox regression (B) model analysis, which included the factors of pathologic stage, histologic grade, CES1, and patient outcomes in the TCGA-BLCA cohort.

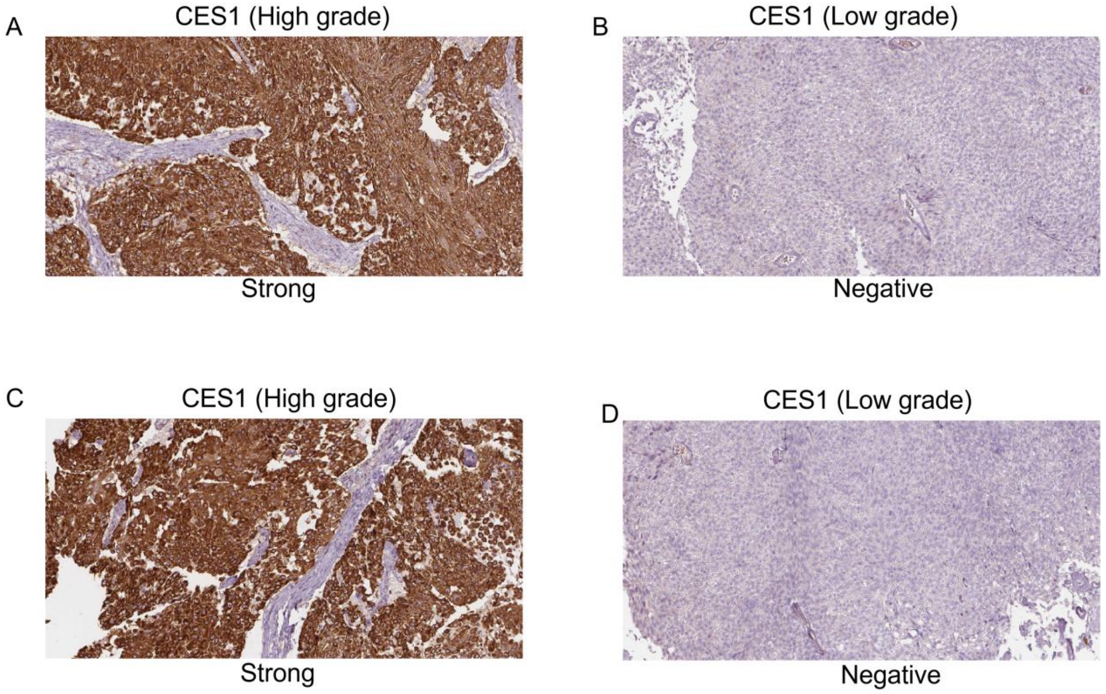

Figure S2 Protein expression levels of CES1 in high-grade (A and C) and low-grade (B and D) urothelial carcinoma based on immunohistochemistry images (HPA). The staining intensity for CES1 was assessed and categorized as negative, low, moderate, or high according to standardized visual evaluation criteria.

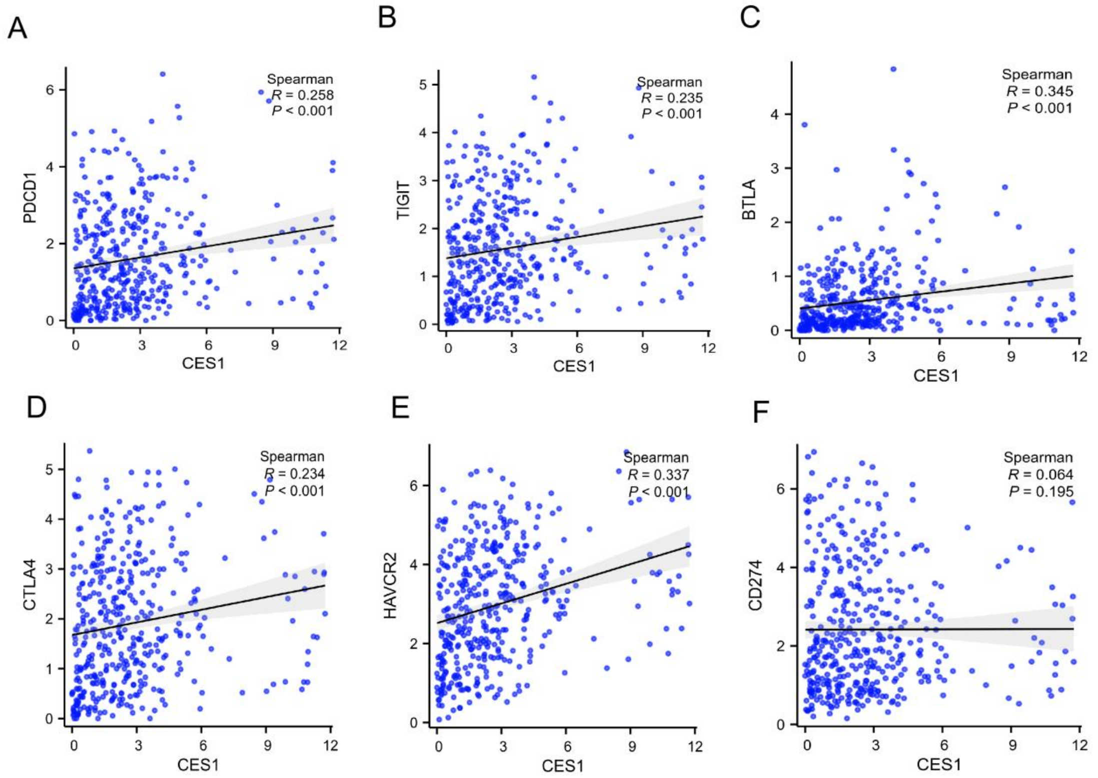

Figure S3. Spearman correlation analysis between CES1 and immune checkpoint gene expression in the TCGA-BLCA cohort.

(A) Correlation between CES1 and PDCD1 (encoding PD-1). (B) Correlation between CES1 and TIGIT. (C) Correlation between CES1 and BTLA. (D) Correlation between CES1 and CTLA4. (E) Correlation between CES1 and HAVCR2 (encoding TIM-3). (F) Correlation between CES1 and CD274 (encoding PD-L1). PD-1, programmed cell death protein 1; TIGIT, T-cell immunoreceptor with Ig and ITIM domains; BTLA, B- and T-lymphocyte attenuator; CTLA4, cytotoxic T-lymphocyte-associated protein 4; TIM-3, T-cell immunoglobulin and mucin domain-containing protein 3; PD-L1, programmed death-ligand 1; R, Spearman's rank correlation coefficient. Statistical significance was defined as $P < 0.05$.

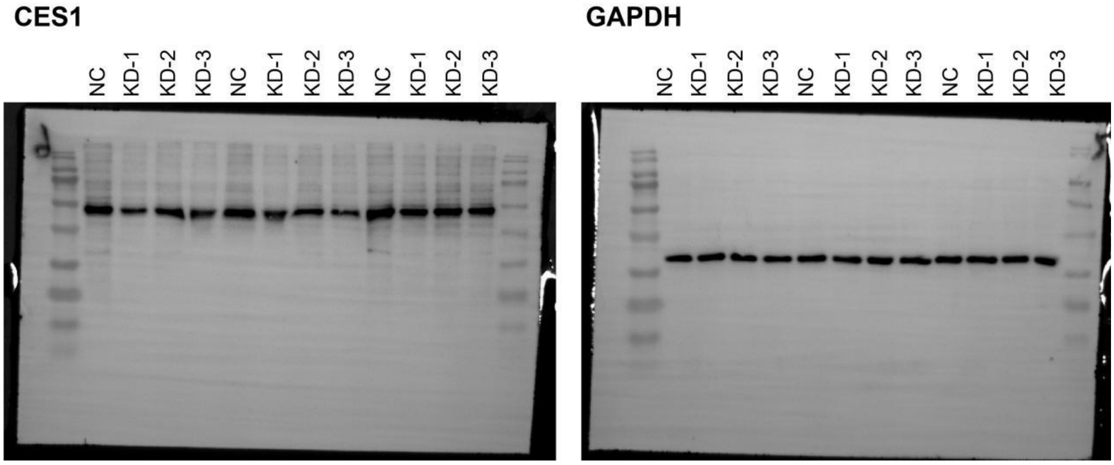

Figure S4 Full length western blots of CES1 knockdown efficiency.

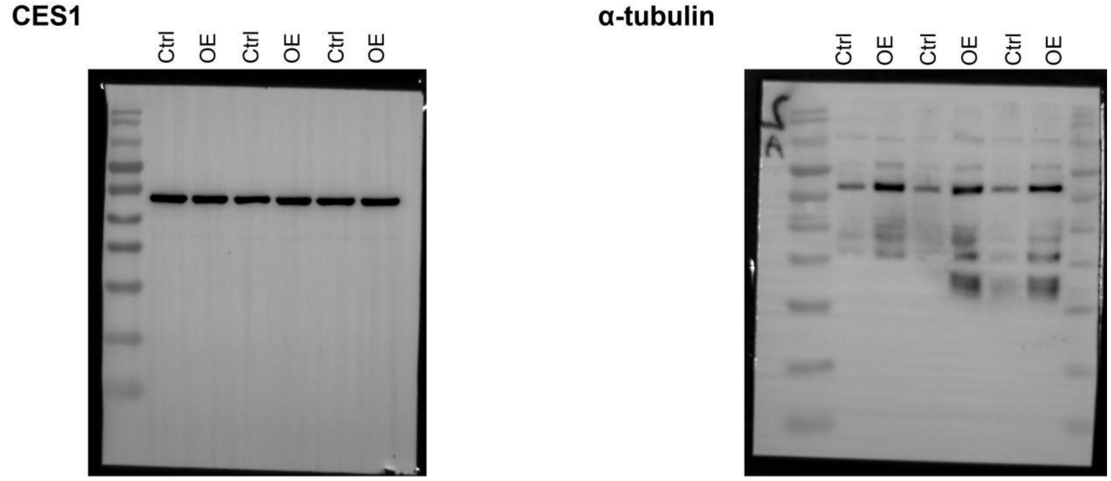

Figure S5 Full length western blots of CES1 overexpression efficiency.
